# Deep learning‐based H&E‐derived risk scores in colorectal cancer: associations with tumour morphology, biology, and predicted drug response

**DOI:** 10.1002/path.70039

**Published:** 2026-02-20

**Authors:** Nic G Reitsam, Xiaofeng Jiang, Junhao Liang, Bianca Grosser, Veselin Grozdanov, Chiara ML Loeffler, Marco Gustav, Tim Lenz, Hannah S Muti, Zunamys I Carrero, Nicholas P West, Philip Quirke, Sebastian Foersch, Moritz Jesinghaus, Wolfram Müller, Tanwei Yuan, Michael Hoffmeister, Hermann Brenner, Jitendra Jonnagaddala, Nicholas J Hawkins, Robyn L Ward, Heike I Grabsch, Bruno Märkl, Jakob N Kather

**Affiliations:** ^1^ Pathology, Faculty of Medicine University of Augsburg Augsburg Germany; ^2^ Bavarian Center for Cancer Research, BZKF Augsburg Germany; ^3^ Else Kroener Fresenius Center for Digital Health Faculty of Medicine, TUD Dresden University of Technology Dresden Germany; ^4^ Department of Thoracic Surgery, Sichuan Clinical Research Center for Cancer, Sichuan Cancer Hospital & Institute, Sichuan Cancer Center University of Electronic Science and Technology of PR China (UESTC) Chengdu PR China; ^5^ State Key Laboratory of Precision Measurement Technology and Instruments, Department of Precision Instrument Tsinghua University Beijing PR China; ^6^ Institute of Pathology Technical University of Munich Munich Germany; ^7^ Department of Neurology Ulm University Ulm Germany; ^8^ Department of Medicine I University Hospital and Faculty of Medicine Carl Gustav Carus, Technische Universität Dresden Dresden Germany; ^9^ National Center for Tumor Diseases Dresden (NCT/UCC), a Partnership Between DKFZ, Faculty of Medicine and University Hospital Carl Gustav Carus TUD Dresden University of Technology, and Helmholtz‐Zentrum Dresden ‐ Rossendorf (HZDR) Dresden Germany; ^10^ Department for Visceral, Thoracic and Vascular Surgery University Hospital and Faculty of Medicine Carl Gustav Carus, Technische Universität Dresden Dresden Germany; ^11^ Pathology and Data Analytics, Leeds Institute of Medical Research at St James's University of Leeds Leeds UK; ^12^ Institute of Pathology University Medical Center Mainz Germany; ^13^ Institute of Pathology Philipps University Marburg and University Hospital Marburg Marburg Germany; ^14^ Pathologie Starnberg Starnberg Germany; ^15^ Division of Clinical Epidemiology and Aging Research German Cancer Research Center (DKFZ), Heidelberg Germany; ^16^ Cancer Prevention Graduate School, German Cancer Research Center (DKFZ) Heidelberg Germany; ^17^ German Cancer Consortium (DKTK), German Cancer Research Center (DKFZ) Heidelberg Germany; ^18^ School of Clinical Medicine, Faculty of Medicine and Health, UNSW Sydney Kensington Australia; ^19^ School of Biomedical Sciences, Faculty of Medicine and Health, UNSW Sydney Kensington Australia; ^20^ Monash University Melbourne Australia; ^21^ Department of Pathology, GROW – Research Institute for Oncology and Reproduction Maastricht University Medical Center+ Maastricht The Netherlands; ^22^ Medical Oncology, National Center for Tumour Diseases (NCT) University Hospital Heidelberg Heidelberg Germany

**Keywords:** colorectal cancer, computational pathology, biomarker, deep learning, whole‐slide image, pathology, gene expression, drug response, histopathology

## Abstract

Over recent years, several deep learning (DL) models have been presented to predict colorectal cancer (CRC) patient survival directly from haematoxylin and eosin (H&E)‐stained routine whole‐slide images (WSIs). Unlike traditional studies that rely on manually defined histopathological features, weakly supervised DL allows training directly on clinical endpoints without prior specification of the model's focus. This offers a unique opportunity to study the tissue morphology underlying these predictions, improving our understanding of disease biology. Here, we present a comprehensive analysis of the clinicopathological features, tumour morphology and biology, as well as gene expression‐based predicted drug response of over 4,000 CRC patients derived from four different international cohorts with available H&E‐inferred DL‐based risk scores (low‐ versus high‐risk as well as absolute risk scores). The results from our study suggest that conventional clinicopathological risk factors, such as grade of differentiation, presence of lymph node metastasis, tumour budding, and percentage of tumour necrosis, are positively associated with DL‐based risk scores. Moreover, CRCs with direct tumour–adipocyte interactions are enriched in the DL‐based high‐risk group. Through detailed morphologic review, we provide comprehensive evidence that direct tumour–adipocyte interaction, a high degree of tumour budding, and poorly differentiated morphology are linked to high DL‐based risk scores. Transcriptomic and genetic subgroups show only limited association with H&E‐derived DL‐based risk scores. Moreover, we present data suggesting that DL‐based low‐ versus high‐risk CRCs may be characterised by differential drug sensitivity. Our study highlights that DL‐based risk scores derived from H&E WSIs not only align with established clinicopathological features but also highlight morphological features, such as tumour–adipocyte interaction, that are not routinely captured by established clinicopathological scoring systems. Moreover, DL‐based risk groups may be associated with a differential treatment response, underlining their potential to guide patient stratification in routine clinical practice. © 2026 The Author(s). *The Journal of Pathology* published by John Wiley & Sons Ltd on behalf of The Pathological Society of Great Britain and Ireland.

## Introduction

Colorectal cancer (CRC) is a biologically and clinically heterogeneous disease requiring appropriate patient stratification to enable targeted and personalised treatment regimens [1, 2, 3]. This heterogeneity is reflected in the tissue morphology captured by routine haematoxylin and eosin (H&E)‐stained pathology slides, which has led to numerous histopathological biomarkers with each of them focusing on different aspects of the tumour and/or microenvironment phenotype [4, 5, 6]. Due to the rapid progress in computational approaches, in particular artificial intelligence‐/deep learning (AI/DL)‐based algorithms, and large‐scale digitisation of tissue slides, different end‐to‐end DL‐based models have been proven to be able to directly predict patient outcomes from whole‐slide images (WSIs) [7, 8, 9, 10, 11]. From a clinical point of view, stage II CRCs are of particular interest in this regard as there is still no perfect biomarker to predict which stage II CRC patients should undergo adjuvant chemotherapy [12]. The potential of DL‐based prognostication models in this scenario has led to the development of commercially available tools such as Histotype Px® Colorectal (https://www.domorediagnostics.com/products) that are approaching integration into clinical care [13]. Since these models are based on tissue morphology, they carry the potential to guide pathologists in better understanding disease biology. We and others have previously shown that tumour–adipocyte interaction is an underappreciated morphological feature in CRC, which not only is linked to a distinct biology and poor prognosis but also has been detected repeatedly by interpretative DL‐based approaches [7, 10, 14, 15, 16, 17, 18, 19]. Nevertheless, such interpretative biological approaches and insights are rare and have not been studied systematically. Despite its routine availability, H&E morphology remains an underutilised source of biological insight with potential relevance for therapy selection. As previously stated, DL‐based models applied to H&E slides are approaching clinical use for prognostication in CRC. Extending these models to guide targeted testing, for example, to identify patients most likely to benefit from emerging antibody–drug conjugates (ADCs) [20] or radioligand therapies, offers a scalable and integrative strategy. Therefore, we investigated whether DL‐based risk stratification from H&E alone captures biological signals relevant to treatment response, positioning morphology‐informed AI as a bridge between pathology and precision oncology.

**Figure 1:**
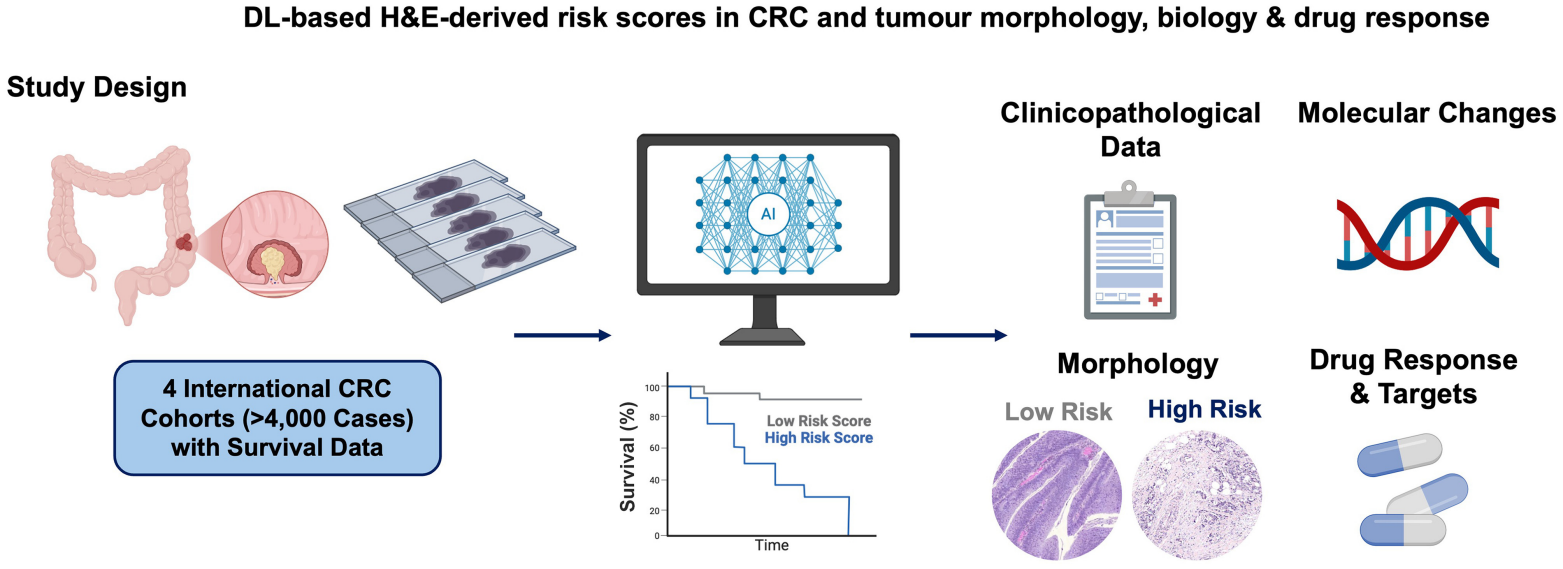
Study design. In our previous publication [7], we established H&E‐inferred DL‐based risk scores through an end‐to‐end DL pipeline. As such end‐to‐end DL‐based risk prediction tools may enter the clinic soon [13], we aimed in the current study to comprehensively analyse the association between end‐to‐end DL‐based risk scores and conventional, established clinicopathological risk factors, tumour morphology, and tumour biology, as well as potential therapeutic vulnerabilities. To do this, we analysed more than 4,000 CRCs from four international cohorts. The figure was initially created in BioRender [Reitsam, N. (2025) https://BioRender.com/lkvd44h] and then modified. CRC, colorectal cancer; H&E, haematoxylin and eosin; DL, deep learning.

Here, we present the results of this study examining the association between DL‐based risk scores and clinicopathological, morphological, and molecular features in four independent CRC cohorts comprising over 4,000 CRCs. The study design is summarised in Figure 1.

## Materials and methods

### Ethical approval

This retrospective study was conducted in accordance with the Declaration of Helsinki [21]. Ethical approval was waived for the underlying DL‐based end‐to‐end prognostication study due to the retrospective nature of the study and the use of anonymised data [7]. Our prior publications may be found in [7, 14, 16, 19].

### Patient cohorts

This retrospective study comprised tissue samples from four different cohorts of over 4,000 patients with resected CRC from Germany (DUESSEL cohort and DACHS cohort), Australia (MCO cohort), and the USA (TCGA‐CRC). The DUESSEL cohort is a case collection of CRC specimens resected with curative intent at the Marien Hospital in Düsseldorf, Germany between January 1990 and December 1995, with the intent of doing research studies [22]. The DACHS study (‘Chancen der Verhütung durch Screening’) is a large population‐based case–control and patient cohort study on CRC from southwestern Germany [23, 24]. The MCO (‘Molecular and Cellular Oncology’) study is a prospective study on more than 1,500 CRC patients treated by curative resection from 1994 to 2010 (https://www.sredhconsortium.org/sredh-datasets/mco-study-whole-slide-image-dataset) [25]. The publicly available TCGA cohorts COAD and READ (colonic and rectal adenocarcinoma) include CRC tissue samples of all stages with multi‐omics characterisation [26]. The clinicopathological characteristics of the cohorts and stratified DL‐based risk status are summarised in supplementary material, Tables S1–S4.

### DL‐based risk scores

The DL‐based prognostic scores used in this study were obtained from a previously published multicentre analysis of end‐to‐end DL models in CRC [7]. In brief, a pretrained histology‐specific encoder, RetCCL [27], was used to extract 2,048‐dimensional feature vectors from image tiles, which were then processed by an attention‐based MIL (attMIL) model [28]. The final risk score was calculated through a weighted aggregation of these tile‐level features. Patients were divided into high‐risk and low‐risk groups by median DL‐based risk scores of the training cohort (median risk score −0.135). Further details may be found in Jiang *et al* [7]. Four different cohorts [DACHS (training and internal testing), MCO from Australia (external testing), TCGA‐CRC (external testing), and DUESSEL (external testing)] were used [7].

Downstream analyses were performed to link these scores to morphology, biology, and predicted drug response.

In Jiang *et al* [7], a rigorous and standardised preprocessing protocol, including removing tiles with artefacts and stain normalisation, was used to ensure high‐quality WSIs. All WSIs were scanned using Leica Aperio scanners (Leica Biosystems, Wetzlar, Germany), thereby mitigating potential image‐quality biases which could affect downstream analyses [7].

### Morphological investigations

We investigated the association between several histopathological biomarkers and morphometric data from prior studies and the DL‐based risk scores [14, 16, 19, 29]. As markers of tumour–adipocyte interaction, we assessed Stroma AReactive Invasion Front Areas (SARIFA) status and tumour‐adipose feature (TAF) status. Whereas SARIFA positivity is defined by direct tumour–adipocyte contact at the invasion front, TAF is characterised by a spatial proximity of tumour cells and adipocytes but not necessarily direct contact [10, 14, 15]. Additionally, we considered the luminal proportion of tumour (PoT) and other morphometrical tissue categories (stroma, lumen, necrosis, vessel, inflammation), measured by so‐called point counting. PoT‐high was defined as > 47% of the area consisting of tumour cells versus PoT‐low with ≤ 47%; this optimized cut‐off was established in a previous study by West *et al* [29]. For TCGA‐CRC, mucinous differentiation was retrieved from the ‘Tumour Type’ metadata (‘colon adenocarcinoma, mucinous type’ and ‘rectal adenocarcinoma, mucinous type’). A two‐tiered grading system (low grade versus high grade; G1/2 versus G3) was applied.

Additionally, we performed a morphological review of the top 20 and bottom 20 cases (highest and lowest DL‐based risk scores) within all cohorts (in total: 160 cases). For each case, we assessed grading (two‐tiered: low grade versus high grade), SARIFA status (if possible; positive versus negative), and the degree of tumour budding [4] [where assessable; from Bd1, Bd2, or Bd3 (Bd1 low, 0–4 buds; Bd2 intermediate, 5–9 buds; Bd2 high, ≥ 10 buds) [4]]. SARIFA status and morphometry data for the DUESSEL cohort were already available from a previous study [19, 29], as was SARIFA status for a subset of TCGA‐CRC [16].

The TCGA cases [26, 30, 31, 32] with corresponding H&E WSIs can be accessed via https://portal.gdc.cancer.gov and/or https://www.cbioportal.org/study/summary?id=coadread_tcga_pub.

### Genetic/transcriptomic subtypes and drug response prediction

Genetic subgroups (COAD‐CIN, COAD‐MSI, COAD‐GS, COAD‐POLE, READ‐CIN, READ‐GS, READ‐POLE, READ‐MSI, NA) for the TCGA‐CRC cases were obtained from cBioPortal [26, 31, 32].

Transcriptomic subtypes were established on normalised gene‐expression counts as described in our previous publication [14] by deploying the R packages CMScaller [33], PDSclassifier [34], and ImmuneSubtypeClassifier [35] (https://github.com/KatherLab/cancer-metadata).

For drug response prediction, the batch‐normalised RNA‐seq data generated with RSEM were obtained from TCGA via cBioPortal [30, 31]. Missing values were replaced with zero counts, and samples with negative counts due to batch correction were not considered. To ensure that training and test data were in the same range, expression data were log_2_‐transformed and a pseudocount of 1 was added (supplementary material, Figure S1). Drug response predictions based on gene expression were then generated using oncoPredict (https://github.com/HuangLabUMN/oncoPredict), with training data from the Genomics of Drug Sensitivity in Cancer (GDSC2) database [GDSC2_Expr (RMA Normalised and Log Transformed) and GDSC2_Res] as well as the Cancer Therapeutics Response Portal (CTRP2) database (CTRP2_Expr, initially not log‐transformed, and CTRP2_Res) [36, 37]. The ‘*calcPhenotype*’ function of oncoPredict was used to generate drug response predictions for 198 (GDSC2) and 545 (CTRP2) drugs with mostly default settings (batch correction: *standardize*; power transformation: *true*; low‐varying gene filter: *0.2*). As TCGA gene expression data are based on RNA‐seq, we applied the *standardize* batch correction to improve compatibility between microarray‐based training data and RNA‐seq testing data (https://rdrr.io/cran/oncoPredict/src/R/CALCPHENOTYPE.R) [37].

### Statistical analyses

*χ* ^2^ tests were used for testing of differences between relative frequencies. Continuous variables were compared using the Wilcoxon rank‐sum test. For the comparison of multiple groups, the Kruskal–Wallis test with *post hoc* pairwise comparisons adjusted for multiple testing was used. Correlations between continuous variables were assessed using Pearson's/Spearman's correlation coefficient (depending on the distribution), with scatter plots visualising linear relationships and with *p* values adjusted for multiple comparisons using the Benjamini–Hochberg method. Linear and logistic regression analyses were performed to assess the association between DL‐based risk scores (absolute or binary) and selected variables; the explained variance was reported using *R* ^2^ and evaluated with *F*‐statistics. Logistic regression models included multiple predictors (SARIFA, T‐stage, nodal status) to estimate the odds ratios (ORs) for DL‐based high‐risk status with corresponding 95% confidence intervals (CIs). To evaluate the discriminatory power of the models, receiver operating characteristic (ROC) curves and area under the curve (AUC) values were calculated. Prognostic performance of DL‐based risk, consensus molecular subtypes (CMS), and pathway‐derived subtypes (PDS), alone and in combination, were assessed using multivariable Cox proportional hazards models for progression‐free survival (TCGA cohort), with five‐fold cross‐validation used to compute mean concordance indices (*C*‐index) and standard deviations across models.

*p* values less than 0.05 were considered statistically significant. *q* values are reported to incorporate multiple testing corrections (using a false discovery rate detection approach). All analyses were performed using R version 4.4.0 (https://cran.r-project.org/bin/windows/base/old/4.4.0/) with relevant packages (survival, survminer, dplyr, tidyr, tidyverse, ggpubr, ggplot2, ggrepel, oncoPredict, CMScaller, PDSclassifier, and ImmuneSubtypeClassifier).

## Results

### H&E‐inferred DL‐based risk scores align with established tissue biomarkers in CRC

**Figure 2:**
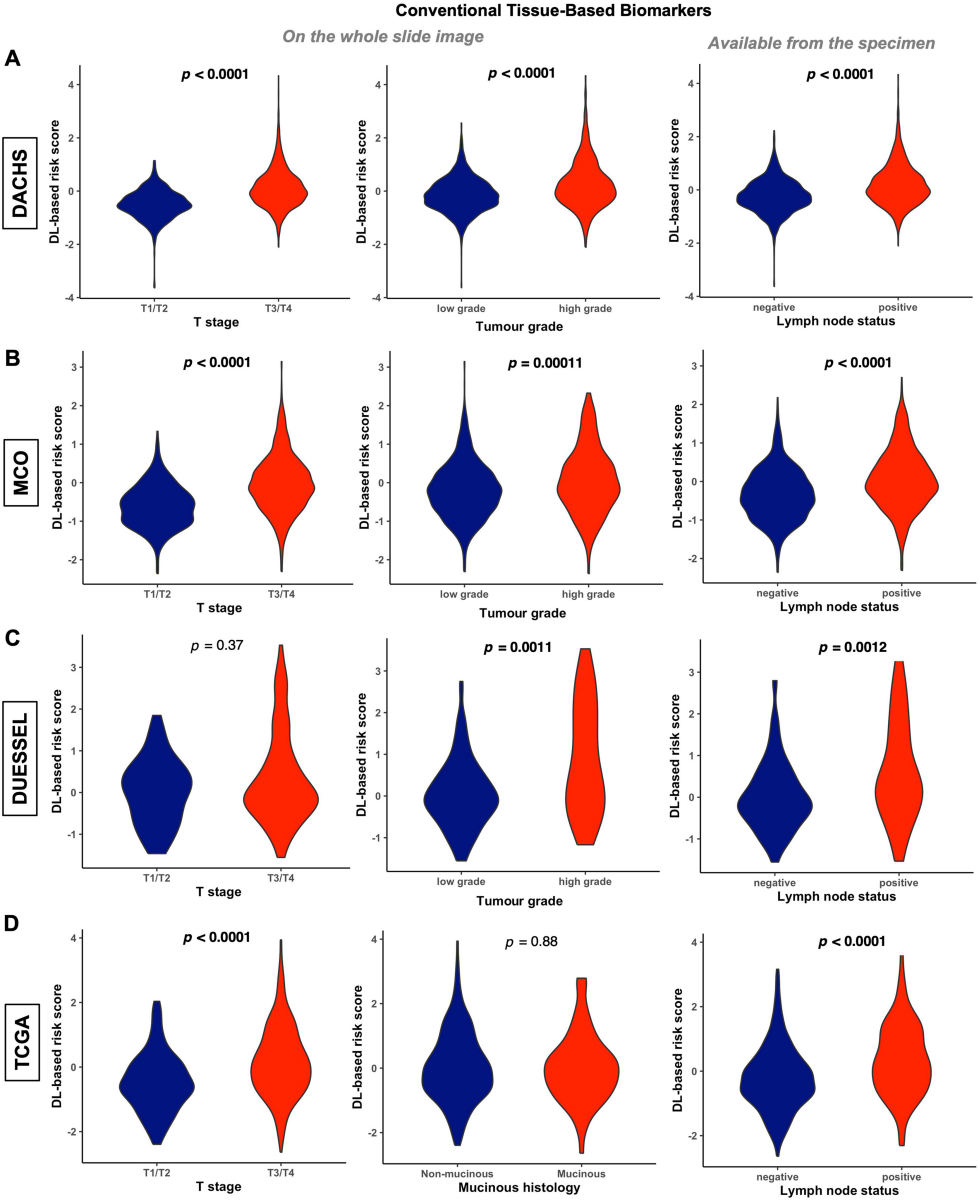
Association between conventional tissue‐based biomarkers and DL‐based prediction scores in four different, international CRC cohorts. (A) DACHS cohort (*n* = 2,281); (B) MCO cohort (*n* = 1,395); (C) DUESSEL cohort (*n* = 159); (D) TCGA COAD and READ cohorts (*n* = 533). As there are no reliable histology grade groups for TCGA available, we included here the comparison between mucinous and non‐mucinous differentiation based on the TCGA metadata, which show no statistically significant differences between DL‐based low‐ and high‐risk groups. *p* values of Wilcoxon test are displayed. Significant *p* values (*p* < 0.05) are highlighted in bold. CRC, colorectal cancer; DL, deep learning; TCGA, The Cancer Genome Atlas.

Although several H&E‐derived DL‐based risk models for CRC have been previously proposed, little is currently known about the association between established conventional tissue biomarkers and these DL‐based risk scores [7, 8, 9, 10, 11]. Therefore, we compared DL‐based risk scores with several known tissue‐based biomarkers in over 4,000 CRCs from four different cohorts. High‐grade morphology (G3), locally advanced tumour stages (T3/T4), and positive lymph node status were associated with higher DL‐based risk scores in almost all cohorts (Figure 2 and supplementary material, Figure S2). Mucinous differentiation was not linked to higher DL‐based risk scores in either the TCGA or the MCO cohort (Figure 2D, middle panel and supplementary material, Figure S3A), whereas in the MCO cohort vascular and perineural invasion were associated with higher DL‐based risk scores (supplementary material, Figure S3B,C).

**Figure 3:**
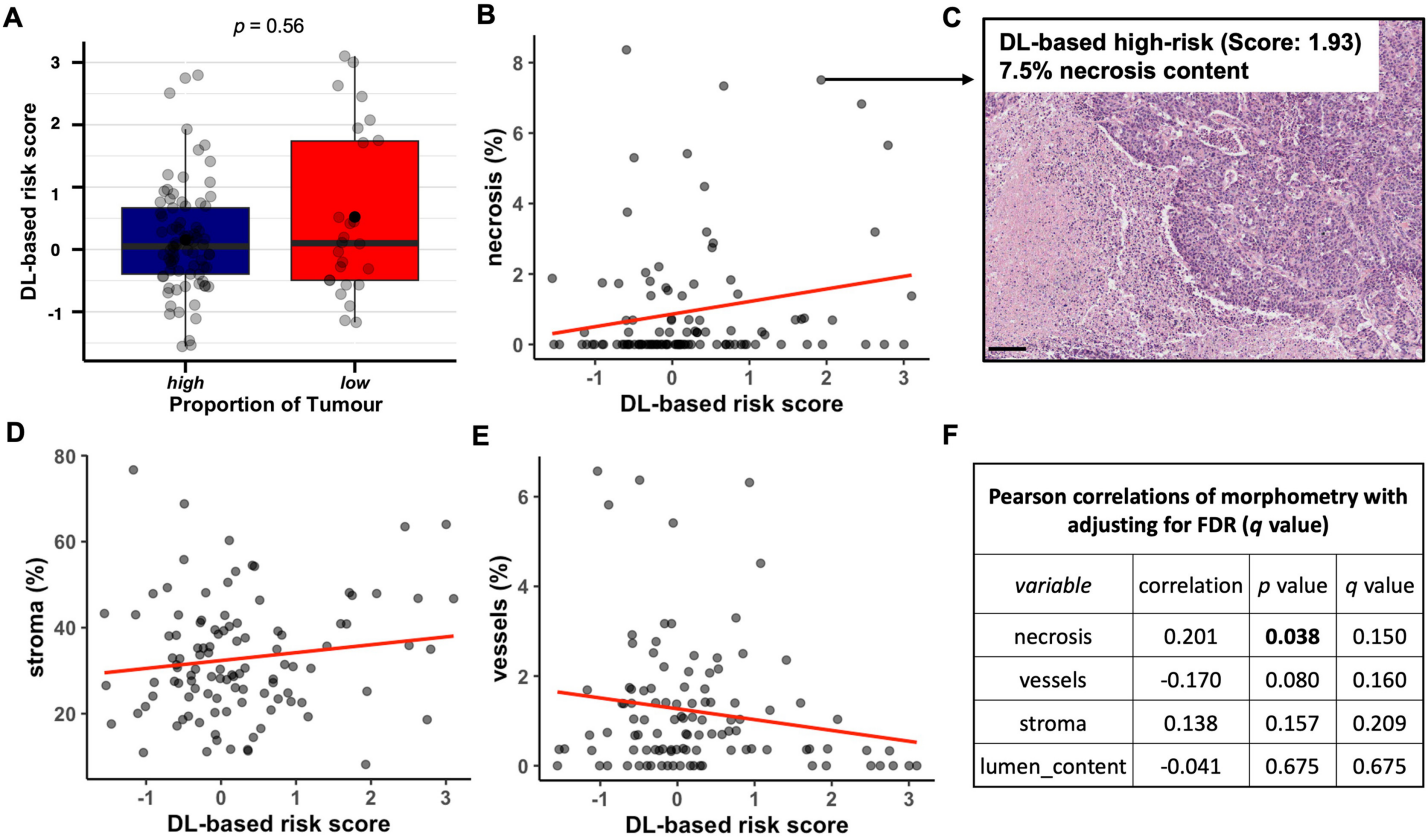
Tissue morphometry and DL‐based risk scores. (A) Comparison of DL‐based risk scores between PoT‐high and PoT‐low CRCs. (B, D, E) Correlation of tumour morphometry [stroma, necrosis, vessel content (area of percentage)] and DL‐based risk scores. (C) H&E histology of CRC with high necrosis content (7.5%) and DL‐based high‐risk status (absolute score: 1.93) displaying extensive necrosis and predominantly solid growth pattern. Scale bar, 100 μm. (F) Summary table of Pearson correlations with *p* values as well as adjusted *q* values using the Benjamini–Hochberg method (FDR). *p* value less than 0.05 is highlighted in bold. In panels B, D, and E, % indicates the percentage areas. The scatterplot for lumen percentage is presented in supplementary material, Figure S4. Morphometry data and prediction scores were available for 107 CRCs of the DUESSEL cohort. CRC, colorectal cancer; DL, deep learning; FDR, false discovery rate; PoT, proportion of tumour.

We next evaluated the correlation between morphometric data and DL‐based risk scores. PoT‐low (e.g. higher stroma content) was not significantly associated with higher DL‐based prediction scores (Figure 3A). Similarly, the proportion of stromal tissue, glandular luminal space, and vasculature did not significantly correlate with the DL‐based risk scores (Figure 3 and supplementary material, Figure S4), whereas the percentage of necrosis showed a trend towards a positive correlation with DL‐based risk scores (*p* = 0.038, *q* = 0.150; Figure 3B,C).

As demographic bias is known to be relevant in computational pathology [38], we analysed the distribution of DL‐based risk scores across ethnic groups in TCGA to assess potential disparities. Our findings showed that the proportion of individuals in the high‐risk category varied across racial groups, with white individuals showing the most balanced distribution between low and high risk. Conversely, Black or African American individuals were disproportionately represented in the DL‐high‐risk group. Fisher's exact test indicated a statistically significant association between race and DL‐based risk (*p* = 0.015), although some subsets included small numbers.

Clinicopathological features between DL‐based low‐ and high‐risk CRCs are summarised in supplementary material, Tables S1–S4.

Interestingly, DL‐high‐risk CRCs exhibited markedly higher metastasis rates in DACHS, MCO, and TCGA (all *p* < 0.001; supplementary material, Table S5), indicating that the DL‐high‐risk group consistently identified patients with a substantially more aggressive course of disease.

### DL‐based risk scores are associated with known high‐risk morphological features

**Figure 4:**
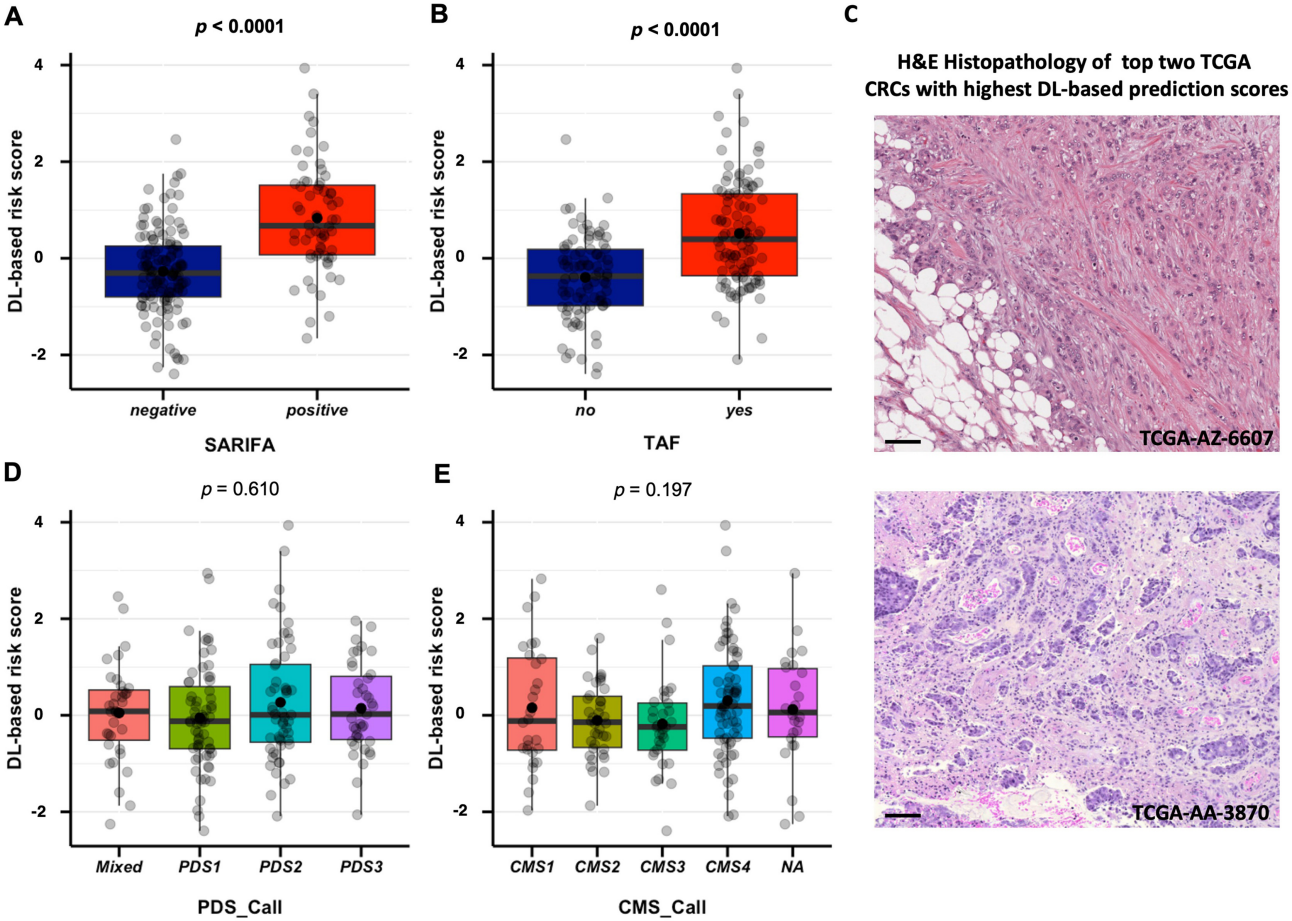
Tumour morphology/biology and DL‐based prediction scores in TCGA‐CRC. (A,B) Comparison of DL‐based risk scores between SARIFA‐positive/negative (A) and TAF‐present/absent (B) CRC patients. (C) H&E histopathology of the top two TCGA‐CRCs with the highest DL‐based prediction scores displaying high‐grade tumour budding, direct tumour–adipocyte interaction (SARIFA positivity), and poor differentiation or micropapillary growth pattern in TCGA‐AA‐3870. (D,E) Comparison of DL‐based risk scores between different PDS (D) and CMS (E) subgroups. Scale bars, 100 μm. Significant *p* values (*p* < 0.05) are highlighted in bold. CMS, consensus molecular subtype; CRC, colorectal cancer; DL, deep learning; H&E, haematoxylin and eosin; PDS, pathway‐derived subtype; SARIFA, Stroma AReactive Invasion Front Areas; TAF, tumour‐adipose feature; TCGA, The Cancer Genome Atlas.

To assess the association between tumour–adipocyte interactions and DL‐based risk scores, we compared the absolute risk scores between SARIFA‐positive and SARIFA‐negative, as well as TAF‐present and TAF‐absent, CRC patients within the TCGA COAD and READ cohorts (*n* = 196 in total). SARIFA‐positive CRC patients as well as CRCs with TAF were characterised by significantly higher DL‐based risk scores (both *p* < 0.0001; Figure 4A–C). In the Düsseldorf cohort, SARIFA‐positive CRCs again exhibited higher DL‐based risk scores (*p* < 0.0001; supplementary material, Figure S5).

We next performed a histopathological review of the 160 cases with the highest or lowest DL‐based risk scores within their cohort (20 high and 20 low per cohort; supplementary material, Figures S6–S10). This histopathological review showed significant enrichment of high‐risk morphological features in CRCs with high DL‐based risk scores. Across all reviewed cases, CRCs with high DL‐based risk scores showed a strong association with high‐grade tumour budding, SARIFA positivity, and high‐grade morphology (all *p* < 0.0001 based on *χ* ^2^ tests; supplementary material, Figure S6).

Interestingly, signet‐ring morphology could be observed in two top DL‐high‐risk TCGA cases (TCGA‐A6‐A565, tenth highest DL‐based prediction score; TCGA‐AY‐6196, 12th highest DL‐based prediction score) as well as two top DL‐high‐risk DACHS cases (14th and 17th highest DL‐based prediction scores; supplementary material, Figure S8). Additionally, three of the top high‐risk cases displayed micropapillary differentiation. The cases with the lowest DL‐based prediction scores were predominantly Bd1, low grade, and SARIFA‐negative, often also with relevant adenoma components and early‐stage/superficially invasive carcinoma (supplementary material, Figures S7 and S10).

Given the strong association between SARIFA positivity, locally advanced CRC (≥ T3), and positive lymph node status with DL‐based high‐risk scores, we performed linear and logistic regression analyses on, in total, *n* = 349 cases from the TCGA cohort and cases with complete data from the Duesseldorf cohort. Linear regression revealed that SARIFA positivity (*β* = 1.15, *p* < 0.0001) and nodal‐positive status (*β* = 0.215, *p* = 0.038) were significantly associated with higher DL‐based risk scores, whereas locally advanced disease in this CRC subset was not (*β* = 0.133, *p* = 0.286). The overall model explained 29.6% of the variance (*R* ^2^ = 0.296), and had a significant *F*‐statistic (*p* < 0.0001). Logistic regression analysis showed that SARIFA positivity was a strong and independent predictor of DL‐based high‐risk status (OR = 8.44, 95% CI 4.46–17.2, *p* < 0.0001), whereas nodal‐positive status showed a trend towards significance (OR = 1.58, 95% CI 0.96–2.61, *p* = 0.073), and locally advanced disease (T3/4) was not significant (OR = 0.77, 95% CI 0.44–1.36, *p* = 0.368). The logistic regression model incorporating SARIFA status, T‐stage, and nodal status demonstrated a moderate ability to distinguish between high and low DL‐based risk status, with an area under the ROC curve of 0.689 (supplementary material, Figure S11), indicating that these features are valuable surrogate markers for DL‐based risk scores but do not capture the full complexity derived from DL‐based risk stratification.

### Beyond H&E: DL‐based risk scores, molecular subtyping, and drug response prediction

Compared with morphological/conventional biomarkers, different gene expression‐based molecular subtypes (CMS/PDS/IS) were not associated with H&E‐inferred DL‐based risk scores (each Kruskal–Wallis *p* value above 0.05; Figure 4D,E and supplementary material, Figures S12 and S13) in the TCGA dataset. To test whether RNA‐based subtypes add prognostic value beyond H&E‐derived DL risk, we performed cross‐validated Cox models in TCGA, where the DL‐based risk score showed the strongest prognostic performance (*C*‐index = 0.607), with no significant improvement from adding CMS or PDS subtypes. CMS and PDS calls did not show prognostic relevance in this subcohort with full data (*n* = 244). These results are presented in supplementary material, Figure S12.

By assessing pairwise differences between DL‐based risk scores and genetically defined subtypes of COAD and READ, we observed higher DL‐based prediction scores in CIN‐COAD and CIN‐READ compared with GS‐COAD (Kruskal–Wallis *p* = 0.00043; *p* values of Dunn's test with Bonferroni correction for both mentioned comparisons < 0.01; supplementary material, Figure S14). In the DACHS and MCO cohorts, the subset of *BRAF*‐mutant/MSS (microsatellite‐stable) CRCs, which is an aggressive CRC subset with a distinct biology [39], displayed significantly higher DL‐based risk scores than other CRCs [*BRAF*‐wild type and *BRAF*‐mutant/MSI (microsatellite‐unstable); supplementary material, Figure S15]. In the larger DACHS and MCO cohorts, MSI status differed significantly by DL‐based risk groups, with an enrichment of MSI CRCs in the DL‐based low‐risk cases (supplementary material, Tables S3 and S4). Nevertheless, the DL‐based risk scores retained their prognostic significance in the MSS and MSI subgroup analysis in most cohorts (supplementary material, Figure S16). When restricted to MSS CRCs, DL‐based risk scores did not differ by mucinous differentiation, which is closely linked to MSI, in the TCGA and MCO cohorts (*p* = 0.69 and *p* = 0.59, respectively; supplementary material, Figure S17).

**Figure 5:**
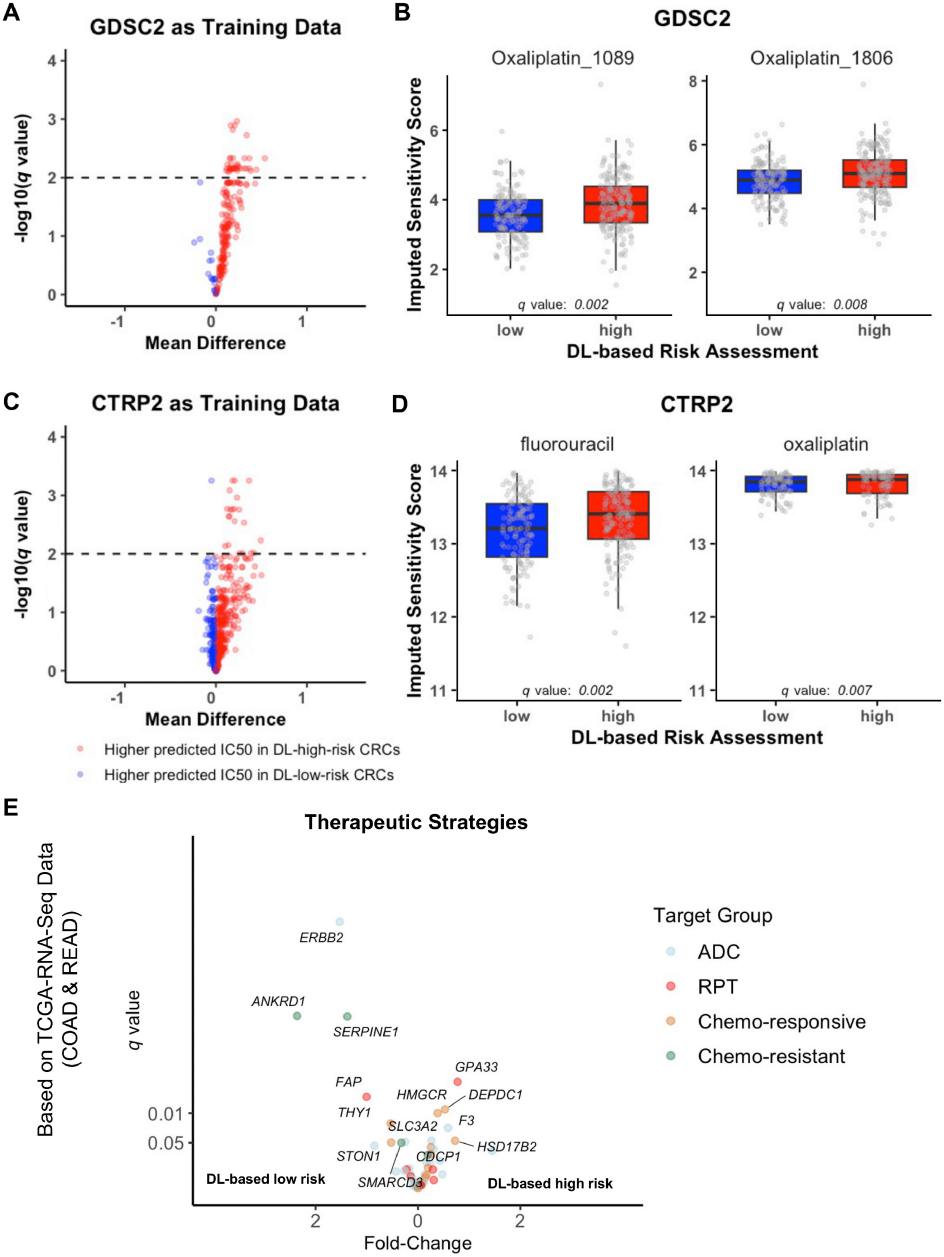
Predicted differential drug response of DL‐based high‐ and low‐risk CRCs. (A,C) Volcano plots illustrating the mean differences in predicted drug response between DL‐based high‐ and low‐risk CRCs, derived from oncoPredict based on TCGA‐CRC (*n* = 326) as test data and GDSC2 or CTRP2 as training data. Each point represents a drug, with the *x*‐axis showing the mean differences in imputed sensitivity scores and the *y*‐axis displaying the −log(*q* value). Drugs with positive mean differences indicate higher predicted resistance in DL‐based high‐risk CRCs, while negative values correspond to higher predicted resistance in DL‐based low‐risk CRCs. The dashed line marks the significance threshold at *q* < 0.01. (B,D) Boxplots comparing imputed sensitivity scores for oxaliplatin_1089 and oxaliplatin_1806/fluorouracil and oxaliplatin between DL‐based high‐ and low‐risk CRCs. Jittered points indicate individual samples, with DL‐based high‐risk CRCs exhibiting significantly higher predicted resistance to both oxaliplatin variants and fluorouracil (*q* values shown for each drug). As these predictions are based on transcriptomic profiles, they highlight potential biological differences in drug response between DL‐inferred risk groups. (E) Differential expression of curated therapeutic targets in DL‐based high‐ versus low‐risk CRCs. Volcano plot showing fold‐change and *q* values (FDR) for a curated panel of genes relevant to antibody–drug conjugates (ADC), radiopharmaceutical therapies (RPT), and chemotherapy resistance or responsiveness. Each point represents a gene, with colour indicating its assigned therapeutic group. Genes with significant differential expression (FDR < 0.05) are labelled. Notably, *GPA33* and *F3* (CD142) were significantly upregulated in DL‐based high‐risk CRCs, suggesting vulnerability to radioligand or ADC‐based therapies, respectively. Conversely, *ERBB2*, *FAP*, and several chemo‐resistance‐associated genes were enriched in DL‐based low‐risk CRCs, indicating distinct, targetable molecular phenotypes across DL‐derived risk groups. oncoPredict is a computational tool that predicts drug response in patient samples by leveraging transcriptomic data and drug sensitivity profiles from cancer cell lines. CRC, colorectal cancer; DL, deep learning; GDSC2, Genomics of Drug Sensitivity in Cancer 2; CTRP2, Cancer Therapeutics Response Portal 2; TCGA, The Cancer Genome Atlas.

Previously, we have shown that DL‐based high‐ and low‐risk CRCs are characterised by a dysregulation of gene expression [7]. Therefore, we applied oncoPredict, a computational tool that derives drug responses based on cell line screening data [36], to assess whether these transcriptomic differences lead to differences in predicted treatment responses. DL‐based high‐risk CRCs indeed displayed differential drug sensitivity (Figure 5A), with a higher predicted resistance for many of the included drugs in the GDSC2 database (36 drugs with *q* < 0.01 in GDSC2). Based on their transcriptomic profile, DL‐based high‐risk CRCs were predicted to be more resistant to oxaliplatin (Figure 5B), a drug commonly used in the treatment of CRCs. We aimed to validate these findings using the CTRP2 database, which includes 545 drugs, and again observed differential drug sensitivity, with increased resistance in DL‐based high‐risk CRCs (Figure 5C; 21 drugs with *q* < 0.01 in CTRP2). Imputed sensitivity scores for DL‐based high‐risk CRCs for oxaliplatin and fluorouracil, both commonly used in the treatment of CRC, were significantly higher compared with DL‐based low‐risk CRCs (Figure 5D).

To further investigate the therapeutic vulnerabilities associated with H&E‐inferred DL‐inferred risk, we curated a therapy‐relevant gene panel including antibody–drug conjugate (ADC) and radiopharmaceutical therapy (RPT) targets, and chemotherapy‐resistant or chemotherapy‐responsive genes (see supplementary material, Table S6). We then assessed the differential expression of genes in this panel between DL‐defined high‐ and low‐risk CRCs using our previously published differential gene expression results [7]. DL‐based low‐risk CRCs may be susceptible to FAP‐ or ERBB2‐directed therapies [all *q* < 0.001, log fold‐change (LFC) > 0.5], which are currently under clinical evaluation. DL‐based high‐risk CRCs exhibited upregulation of GPA33 (*q* < 0.001, LFC = 0.387) and F3 (*q* = 0.017, LFC = 0.293), targets currently under development for radioligand therapy and ADC payload delivery, respectively (Figure 5E).

## Discussion

In recent years, several DL‐based risk stratification models developed on routine H&E slides have been proposed to predict CRC patient outcomes [7, 8, 9, 10, 11]. Some of these models may soon enter clinical practice [13], offering a data‐driven, yet still morphology‐based approach to predicting patient outcomes. Such models have relevance for patients with stage II CRC, where decisions regarding the use of adjuvant chemotherapy remain challenging [12].

We have shown that DL‐based high‐risk scores align with established prognostic markers such as poor differentiation, nodal involvement, and invasion front features such as SARIFA [6, 16, 17, 19, 40, 41, 42], or other morphologic features such as necrosis percentage. Notably, some of these associations (e.g. lymph node status) involved information not directly visible on the H&E slides used by the model, suggesting that the risk score integrates subtle surrogate patterns. The association with a spatially restricted feature such as tumour–adipocyte interaction (SARIFA/TAF) suggests that the model can detect localised patterns relevant for risk stratification. Several other DL‐based studies have also identified tumour–adipocyte interaction as a distinct biological phenomenon linked to a poor prognosis [7, 10, 18, 43, 44]. The positive correlation with tumour necrosis, another adverse histological feature [45], further supports the notion that DL‐based risk scores capture biology already recognised as unfavourable, which enhances their interpretability. Unlike human pathologists who interpret full histological slides with varying levels of resolution, these models are mostly tile‐based, as in our study, analysing integrated features from several hundred cells at once: hence, not on a full slide and not on a single‐cell level.

Interestingly, among the cases with the highest DL‐prediction scores (e.g. those with the poorest predicted prognosis), some displayed signet‐ring or micropapillary differentiation. Both histological CRC subtypes are known to be associated with a poor prognosis and can be regarded as high‐risk morphological subtypes [46, 47]. This observation may provide an initial indication that the model's predictions may capture features linked to these aggressive morphological subtypes; however, given the low case numbers, this finding should be interpreted with caution, and confirmation in specifically annotated cohorts will be required.

Although the DL‐based risk model was trained in a weakly supervised end‐to‐end manner, our study provides extensive pathology‐based validation, showing that high‐ and low‐risk groups strongly correspond to established and emerging histomorphological risk factors, including tumour budding, differentiation grade, or tumour–adipocyte interaction.

Although transcriptomic subgroups of CRC are of prognostic value and are known to be associated with certain morphological features [1, 48, 49], DL‐based risk scores showed little overlap with transcriptomic subgroups. This suggests that H&E‐derived DL models capture partly independent prognostic signals but also highlights the potential for multimodal integration of histology and molecular data [50, 51, 52].

As Black or African American individuals are underrepresented in TCGA, the observed racial disparities in DL‐based risk scores, with these individuals slightly overrepresented in the DL‐based high‐risk group, highlights the need for diverse, well‐balanced training datasets to ensure equitable AI‐driven risk stratification [38].

With the potential introduction of end‐to‐end DL‐based prognostication models in the clinic, DL‐based risk groups/scores may become available for CRC patients in routine care. Beyond risk stratification, the ultimate goal is to guide treatment decisions. Using oncoPredict, which infers drug sensitivity from transcriptomic data [36], we found that DL‐based high‐risk CRCs exhibit a pattern of predicted resistance to multiple therapies, consistent with the observed dysregulation of gene expression in DL‐based high‐risk versus DL‐based low‐risk CRCs [7]. DL‐based high‐risk CRCs were predicted to be more resistant to oxaliplatin. We previously described an upregulation of epithelial–mesenchymal transition (EMT)‐associated gene expression pathways in DL‐based high‐risk CRCs [7], which is known to be linked to oxaliplatin resistance [53]. These findings highlight that the molecular profiles underlying H&E‐DL‐based high‐risk CRCs may also influence drug sensitivity. However, whether these predictions translate into actual treatment responses in patients remains an open question. Since DL‐based risk scores are derived from routinely available H&E histopathology, integrating them into prospective clinical trials provides a unique opportunity to assess whether these models can move beyond prognostication to predict real‐world treatment responses.

We further assessed the differential expression of ADC‐ and RPT‐targets between DL‐defined risk groups, revealing distinct therapeutic vulnerabilities. These findings suggest that DL‐based risk stratification not only could serve a prognostic role but also function as a pre‐screening tool to prioritise patients for target validation assays (e.g. via IHC), thereby repositioning H&E‐based DL as a triaging system to guide therapeutic decision‐making. While *post hoc* explainable AI methods (e.g. GradCAM) highlight areas of model focus but remain a subject of discussion [54], our sapproach complements this by correlating DL‐based risk scores with clinicopathological features, transcriptomic subgroups, and predicted drug responses, providing an independent layer of biological validation. Although associative, these correlations help to build confidence in the model's predictions by revealing links to established cancer biology.

This study has several important limitations. First, it represents a retrospective secondary analysis of previously established DL‐based risk scores. Second, most findings are correlative in nature, and mechanistic explanations remain to be established. Third, the drug response predictions are inferred from transcriptomic profiles and have not yet been clinically validated. Finally, although we observed signals for rarer histological subtypes, systematic morphological annotation was not performed across all cases. Therefore, these observations should be considered hypothesis‐generating and require follow‐up in dedicated studies.

Although the included cohorts span different time periods, DL‐based risk scores derived from Jiang *et al* [7] have previously been shown to generalise robustly across all cohorts, and in our study, they remained prognostic within molecularly defined subgroups (MSI/MSS). As the model relies on histomorphological features, temporal differences in cohort collection are unlikely to bias downstream analyses. Variability in stain appearance can be further mitigated by standard stain normalisation approaches, while the core morphological patterns remain preserved.

Our DL model was intentionally trained on primary colorectal carcinomas, and its biological signal therefore reflects morphology present at the initial tumour site. Whether the same risk‐associated features persist, evolve, or diverge in metastatic lesions remains an open question. Dedicated models trained on metastatic tissue, or analyses in future cohorts with paired primary–metastasis slides, will be required to determine whether risk stratification can be reliably extended to metastatic lesions or whether metastasis‐specific morphological programmes merit separate modelling.

A major strength of this study is the use of one of the largest available multi‐cohort collections of CRC patients (> 4,000 cases) with harmonised DL‐based risk scores, which allowed consistent analyses across diverse international datasets. The integration of histopathology established clinicopathological features, and transcriptomic drug‐response predictions provide a comprehensive, multimodal perspective.

## Conclusions

Our study demonstrates that H&E‐inferred DL‐based risk scores not only align with established tissue biomarkers in CRC, thereby building confidence in their validity, but also capture additional morphological features and are associated with predicted differences in drug response. This suggests that such models can provide scalable, reproducible, and potentially more integrative assessments than conventional pathology alone. Future studies should validate these models in prospective trials, integrate them with multimodal data, and assess how DL‐inferred risk profiles might guide personalised treatment decisions in clinical practice.

## Author contributions statement

NGR and JNK conceived the study and designed the experiments. NGR performed the analysis. JL, XJ, BG, VG, CMML, MG, TL, HSM, ZIC, NPW, PQ, WM, TY, MH, HB, JJ, NJH, RLW and HIG contributed to patient cohorts, clinical datasets and data interpretation. BG, MJ, SF, HIG, PQ, NJH and BM provided expert pathological interpretation. BM and JNK supervised the research. NGR prepared the manuscript with input from all authors.

## Supporting information


**Figure S1.** Expression values after preprocessing for the oncoPredict model
**Figure S2**. Association between DL‐based prediction scores and detailed T‐stage/lymph node status in TCGA‐CRC
**Figure S3**. Comparison between different histopathological features and DL‐based risk scores in the MCO cohort
**Figure S4**. Scatter plot for the correlation between DL‐based risk scores and lumen percentage
**Figure S5**. SARIFA status and DL‐based H&E‐derived risk scores in the DUESSEL‐CRC cohort
**Figure S6**. Histomorphological review of the top and bottom cases with the highest and lowest DL‐based risk scores
**Figure S7**. H&E histopathology of CRC cases with the top and bottom DL‐based prediction scores in the DUESSEL‐CRC cohort
**Figure S8**. Signet‐ring morphology in TCGA is associated with high‐risk scores
**Figure S9**. H&E histopathology of CRC cases with the highest DL‐based H&E‐derived risk scores in the MCO and DACHS cohorts
**Figure S10**. Histopathology of TCGA‐CRC cases with the lowest DL‐based prediction scores
**Figure S11**. AUC for logistic regression predicting binary DL‐based risk status from SARIFA status, T‐stage and lymph node status as conventional biomarkers
**Figure S12**. Association and prognostic value of RNA‐based molecular subtypes relative to DL‐based risk scores in a TCGA subcohort
**Figure S13**. Pan‐cancer immune subtypes and DL‐based prediction scores in TCGA‐CRC
**Figure S14**. DL‐based risk prediction scores in different genetically defined CRC subgroups of TCGA
**Figure S15**. DL‐based H&E‐inferred risk scores and their association with the aggressive subgroup of *BRAF*‐mutant/MSS CRCs
**Figure S16**. Survival curves of CRC patients stratified by MSI/MSS status
**Figure S17**. DL‐based H&E‐inferred risk scores and their association with mucinous histology in the MSS subgroup
**Table S1**. Relationship between DL‐based risk group and clinicopathological features (DUESSEL)
**Table S2**. Relationship between DL‐based risk group and clinicopathological features (TCGA‐COAD/READ)
**Table S3**. Relationship between DL‐based risk group and clinicopathological features (DACHS)
**Table S4**. Relationship between DL‐based risk group and clinicopathological features (MCO)
**Table S5**. Relationship between DL‐based risk group and metastasis
**Table S6**. Potential antibody–drug conjugate (ADC) and radiopharmaceutical therapy (RPT) targets in colorectal cancer

## Data Availability

The Molecular and Cellular Oncology (MCO) study dataset is available through the Secure Research Environment for Digital Health Consortium (https://www.sredhconsortium.org) and was used with approvals. Data from The Cancer Genome Atlas Colorectal Cancer (TCGA) study are publicly available at https://portal.gdc.cancer.gov/. All codes for the DL‐based risk classifier have been previously published (Jiang *et al*, *Lancet Digital Health* 2024) and are open source and available at https://github.com/KatherLab/marugoto and https://github.com/KatherLab/deepmed. Trained models are available at https://github.com/KatherLab/crc-models-2022. The remaining data were provided by the corresponding study investigators, and specific data‐sharing policies can be found in the corresponding original publications.
