# Supplementary material for: Deep learning‐based H&E‐derived risk scores in colorectal cancer: associations with tumour morphology, biology, and predicted drug response
Source: J Pathol. 2026 Feb 20;269(1):112–24. doi: 10.1002/path.70039 (PMC13050814; doi:10.1002/path.70039)
Supplement: Supplementary file 1 — Figure S1. Expression values after preprocessing for the oncoPredict model Figure S2. Association between DL‐based prediction scores and detailed T‐stage/lymph node status in TCGA‐CRC Figure S3. Comparison between different histopathological features and DL‐based risk scores in the MCO cohort Figure S4. Scatter plot for the correlation between DL‐based risk scores and lumen percentage Figure S5. SARIFA status and DL‐based H&E‐derived risk scores in the DUESSEL‐CRC cohort Figure S6. Histomorphological review of the top and bottom cases with the highest and lowest DL‐based risk scores Figure S7. H&E histopathology of CRC cases with the top and bottom DL‐based prediction scores in the DUESSEL‐CRC cohort Figure S8. Signet‐ring morphology in TCGA is associated with high‐risk scores Figure S9. H&E histopathology of CRC cases with the highest DL‐based H&E‐derived risk scores in the MCO and DACHS cohorts Figure S10. Histopathology of TCGA‐CRC cases with the lowest DL‐based prediction scores Figure S11. AUC for logistic regression predicting binary DL‐based risk status from SARIFA status, T‐stage and lymph node status as conventional biomarkers Figure S12. Association and prognostic value of RNA‐based molecular subtypes relative to DL‐based risk scores in a TCGA subcohort Figure S13. Pan‐cancer immune subtypes and DL‐based prediction scores in TCGA‐CRC Figure S14. DL‐based risk prediction scores in different genetically defined CRC subgroups of TCGA Figure S15. DL‐based H&E‐inferred risk scores and their association with the aggressive subgroup of BRAF‐mutant/MSS CRCs Figure S16. Survival curves of CRC patients stratified by MSI/MSS status Figure S17. DL‐based H&E‐inferred risk scores and their association with mucinous histology in the MSS subgroup Table S1. Relationship between DL‐based risk group and clinicopathological features (DUESSEL) Table S2. Relationship between DL‐based risk group and clinicopathological features (TCGA‐COAD/READ) Table S3. Relationship between DL‐ [file PATH-269-112-s001.docx]

**Deep learning-based H&E-derived risk scores in colorectal cancer: associations with tumour morphology, biology, and predicted drug response**

NG Reitsam *et al. J Pathol* <https://doi.org/10.1002/path.70039>

**Supplementary Figures S1–S17**

**Supplementary Tables S1–S6**

**
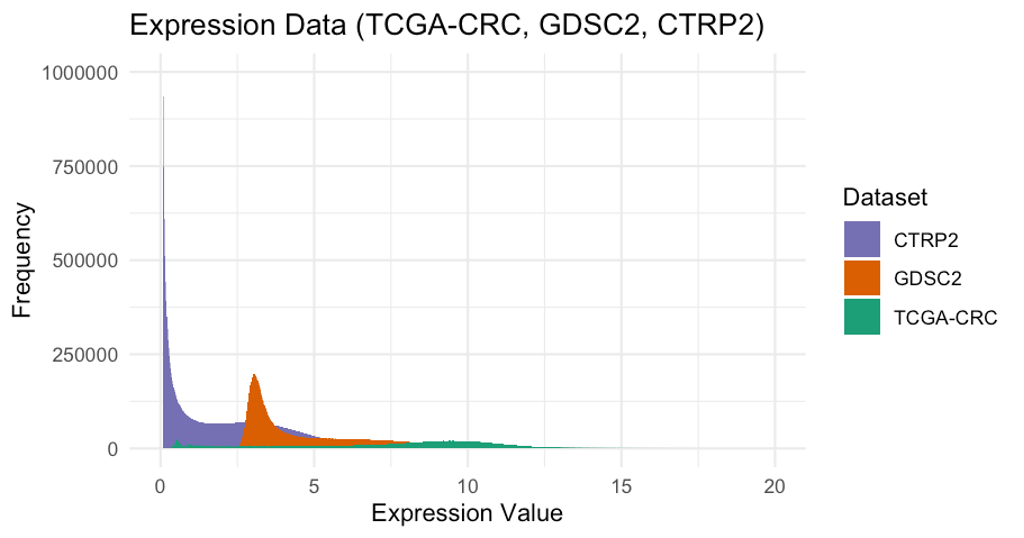
**

**Figure S1. Expression values after preprocessing for the *oncoPredict* model.** To ensure that our test (TCGA-CRC) and training data (GDSC2_Expr/CTRP2_Expr) displayed comparable ranges of expression values – despite originating from different platforms (RNA-seq versus microarray data), we applied appropriate preprocessing steps and used RMA- and log-normalised GDSC2 counts. Batch effects arising from platform differences were addressed using the ‘standardize’ option in the ‘calcPhenotype’ function from *oncoPredict*. A total of 16,394 (GDSC2)/18,370 (CTRP2) gene identifiers overlapped between the two expression matrices, ensuring a broad representation of genes in the model. 5,477/5,692 low-variability genes were filtered out prior to modelling. The final dataset contained 805 samples in GDSC2, 813 in CTRP2, and 365 samples in TCGA-CRC post-preprocessing. Of these, 326 TCGA-CRC samples had available DL-based risk scores. The ratio of DL-based high- and low-risk samples was comparable between the full cohort (279/254 = 1.1) and the subset with risk scores (180/146 = 1.2), supporting the representativeness of the analysed subset.

**
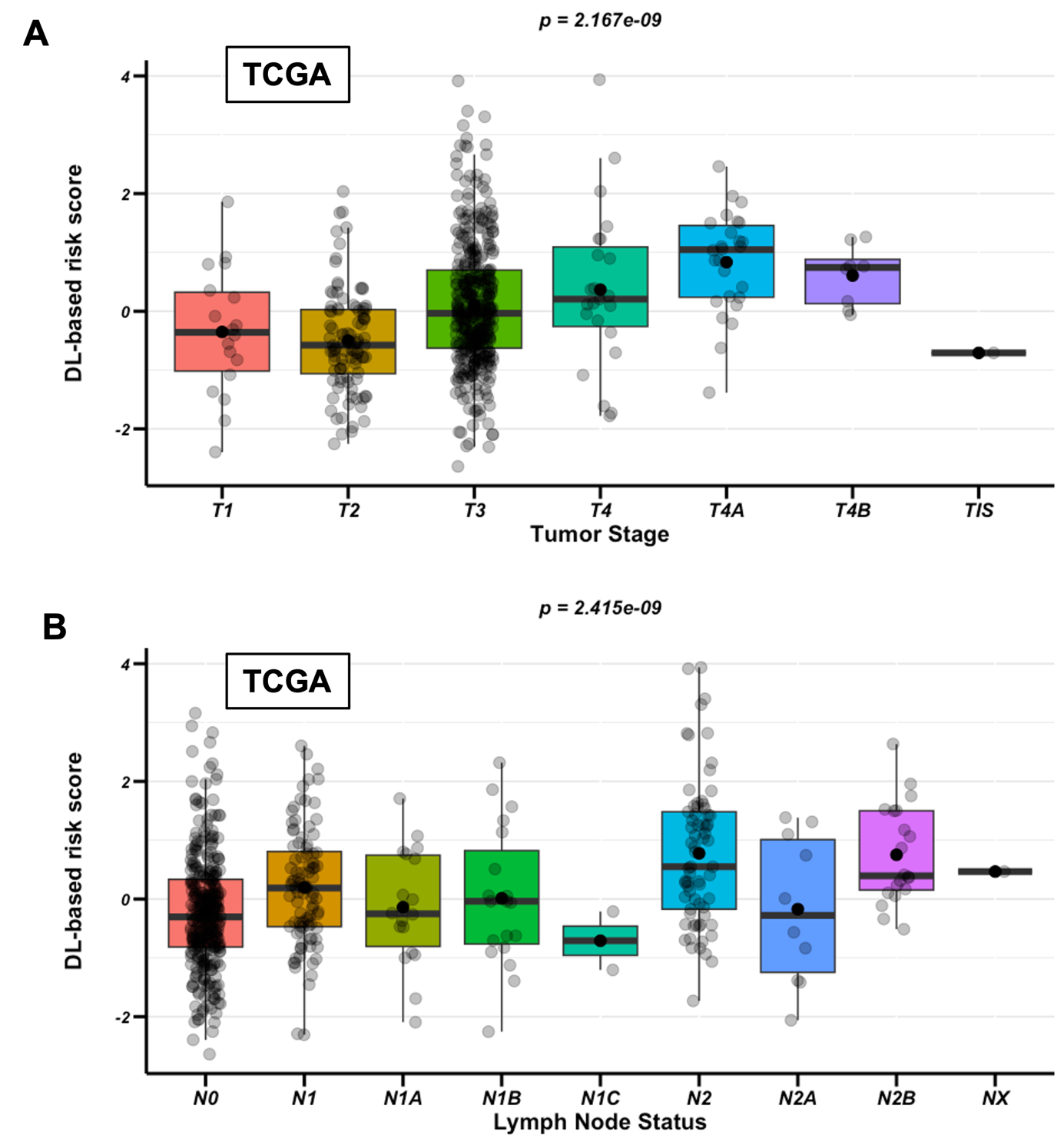
**

**Figure S2. Association between DL-based prediction scores and detailed T-stage/lymph node status in TCGA-CRC.** (A, B) There is an association between higher DL-based risk scores, tumour stage, and lymph node metastasis in TCGA-CRC. The positive association between DL-based risk scores and binary nodal status (positive or negative) is visualized in Figure 2 for all cohorts. *p* values of Kruskal–Wallis tests are displayed. CRC, colorectal cancer; DL, deep learning.


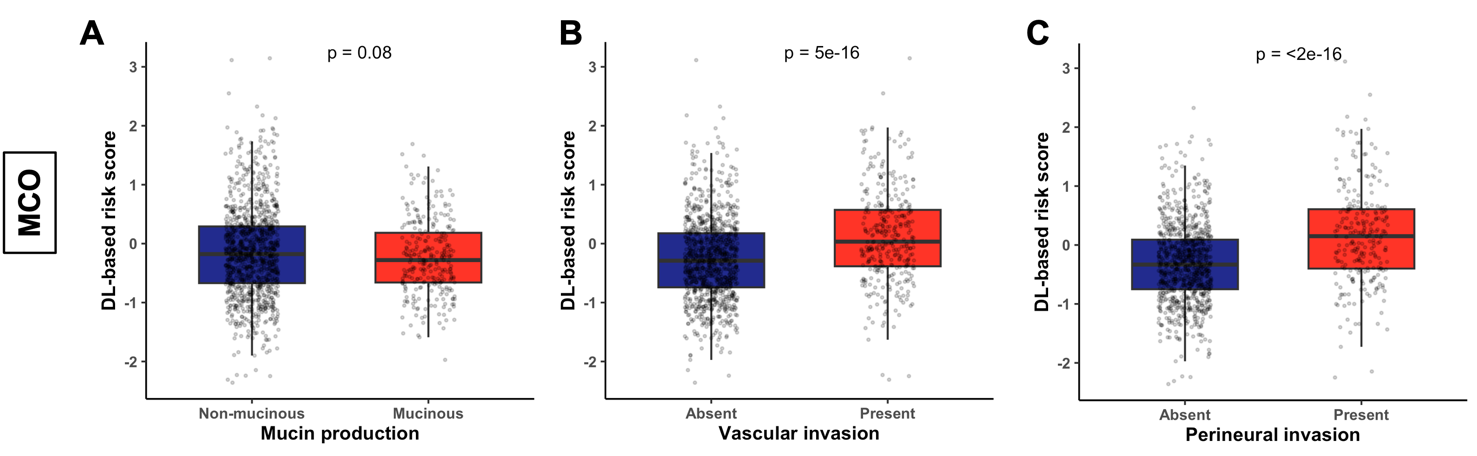
**Figure S3. Comparison between different histopathological features and DL-based risk scores in the MCO cohort.** (A–C) Whereas the presence of vascular invasion (B) and perineural invasion (C) are both significantly associated with higher DL-based H&E-inferred risk scores (both *p <*0.0001), DL-based risk scores did not differ significantly by mucinous differentiation (A). *p* values of Wilcoxon tests are displayed. DL, deep learning.


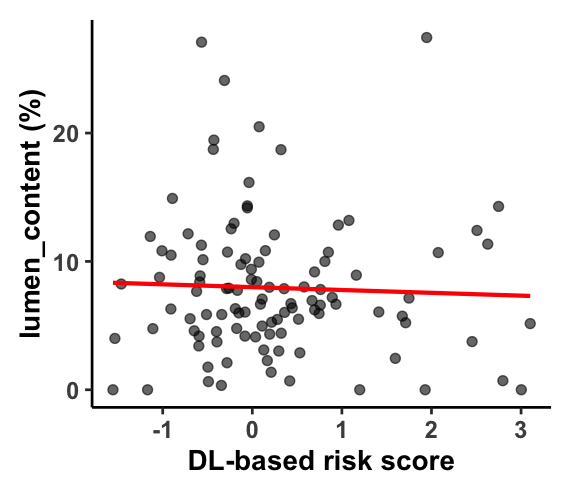
**Figure S4. Scatter plot for the correlation between DL-based risk scores and lumen percentage.** Each dot represents an individual case, with the red line indicating the linear regression fit. The Pearson correlation coefficient was −0.041, with a *p* value of 0.675, indicating no significant correlation between DL-based risk scores and lumen content percentage. DL-based risk scores and stromal morphometry were available for 107 CRC patients of the DUESSEL cohort. CRC, colorectal cancer; DL, deep learning.


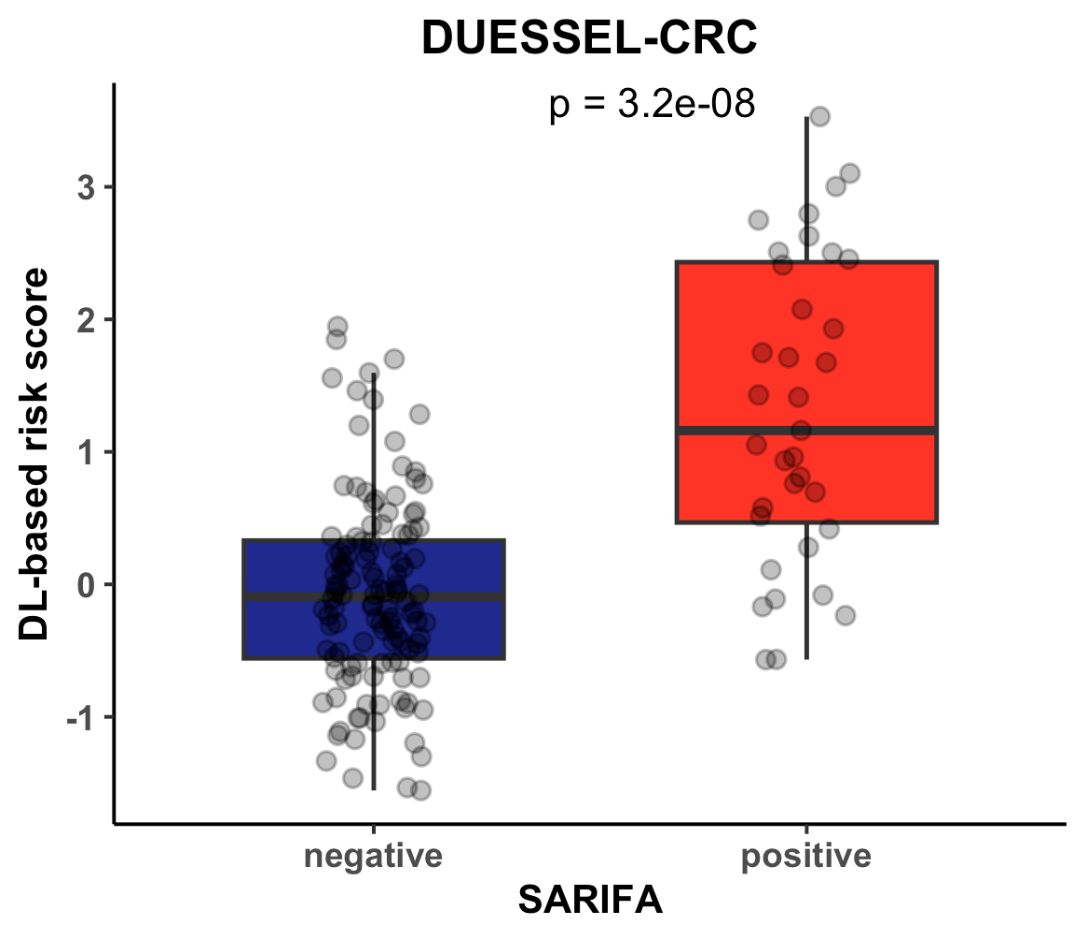
**Figure S5. SARIFA status and DL-based H&E-derived risk scores in the DUESSEL-CRC cohort.** In the DUESSEL cohort, SARIFA-positive CRC patients show significantly higher DL-based H&E-inferred risk scores than do SARIFA-negative CRC patients. *p* values of Wilcoxon tests are displayed. CRC, colorectal cancer; DL, deep learning; SARIFA, Stroma AReactive Invasion Front Areas.


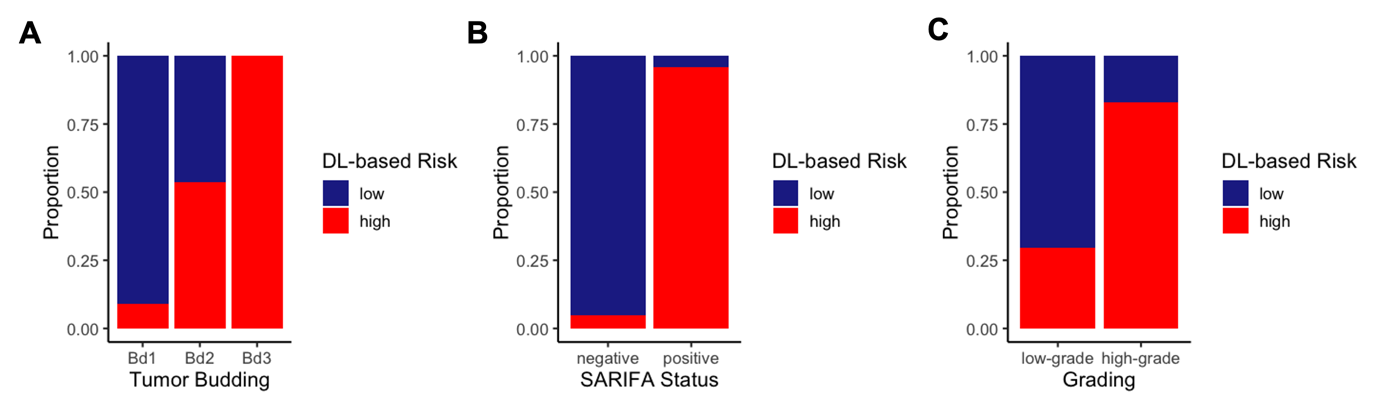


**Figure S6. Histomorphological review of the top and bottom cases with the highest and lowest DL-based risk scores.** (A–C) Proportions of tumour budding grade (A), SARIFA status (B), and tumour grading (C) stratified by DL-based risk group based on the morphological review of the top 20/bottom 20 CRC cases with the highest/lowest DL-based risk scores for each cohort (*n* in total = 160). High DL-risk CRCs were significantly enriched for high-grade tumour budding (*p* < 0.0001), SARIFA positivity (*p* < 0.0001), and high-grade differentiation (*p* < 0.0001). *p* values of *χ*^2^ tests are provided. CRC, colorectal cancer; DL, deep learning; SARIFA, Stroma AReactive Invasion Front Areas.

**
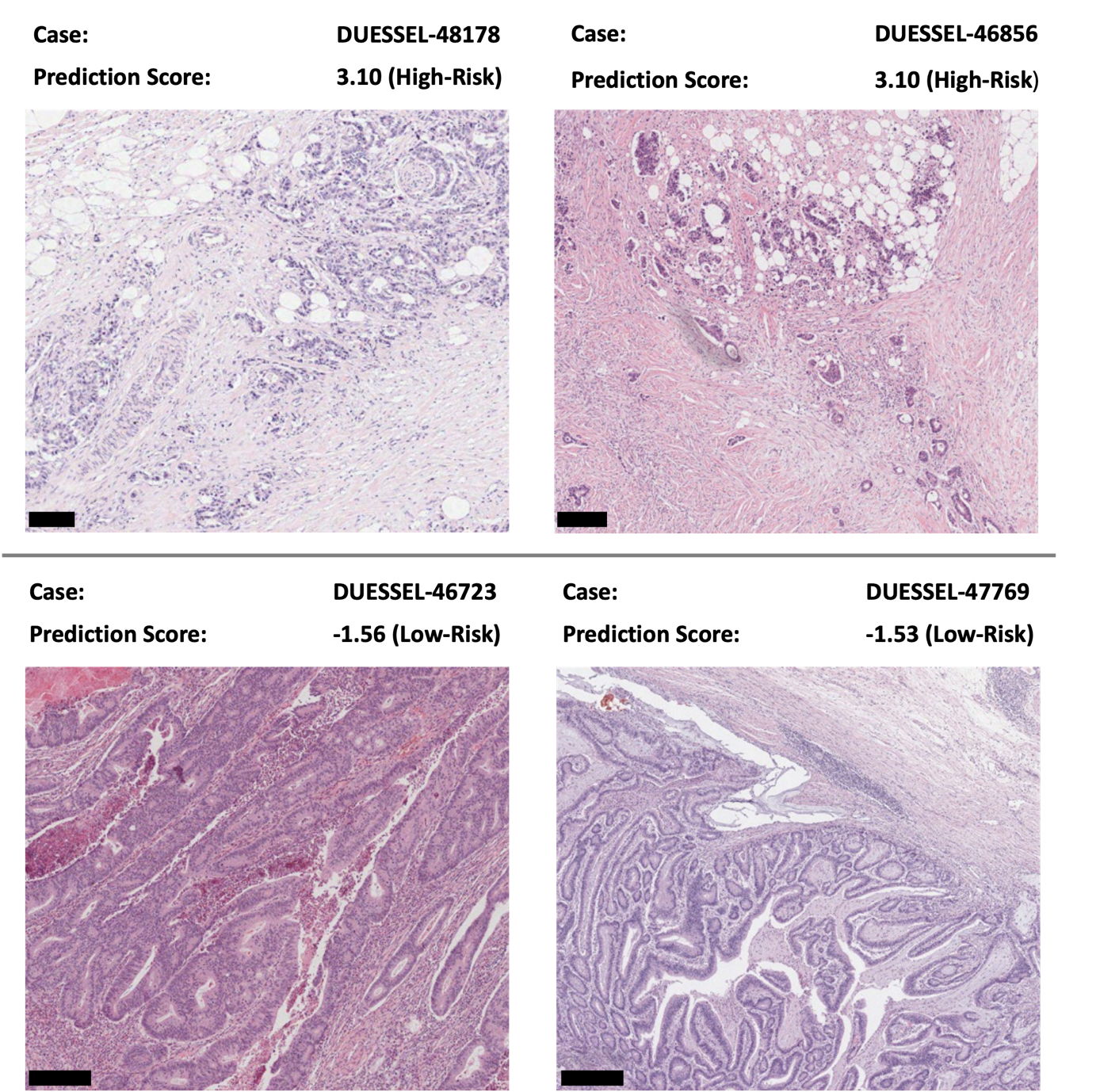
**

**Figure S7. H&E histopathology of CRC cases with the top and bottom DL-based prediction scores in the DUESSEL-CRC cohort.** Whereas the DL-based high-risk cases in the upper panels display high-risk morphological features, such as SARIFA positivity or high tumour budding, the DL-based low-risk cases in the lower panels show low-grade morphology. Scale bars, 100 µm in upper left panel and 200 µm the remaining panels. CRC, colorectal cancer; DL, deep learning; SAR,IFA, Stroma AReactive Invasion Front Areas.

**
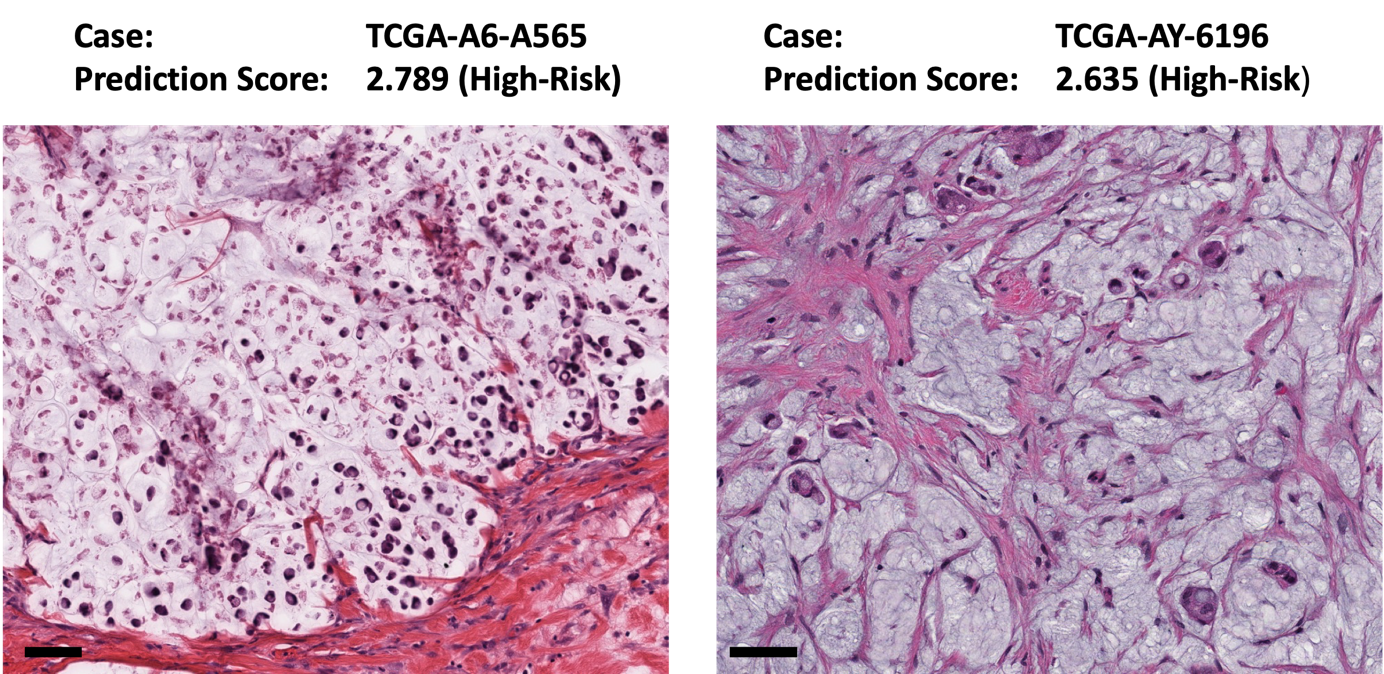
**

**Figure S8. Signet-ring morphology in TCGA is associated with high-risk scores.** The top 20 cases with the highest DL-based risk prediction scores (high-risk) in TCGA also included two cases with a signet-ring cell morphology with mucinous differentiation. Scale bars, 100 µm. DL, deep learning; TCGA, The Cancer Genome Atlas.


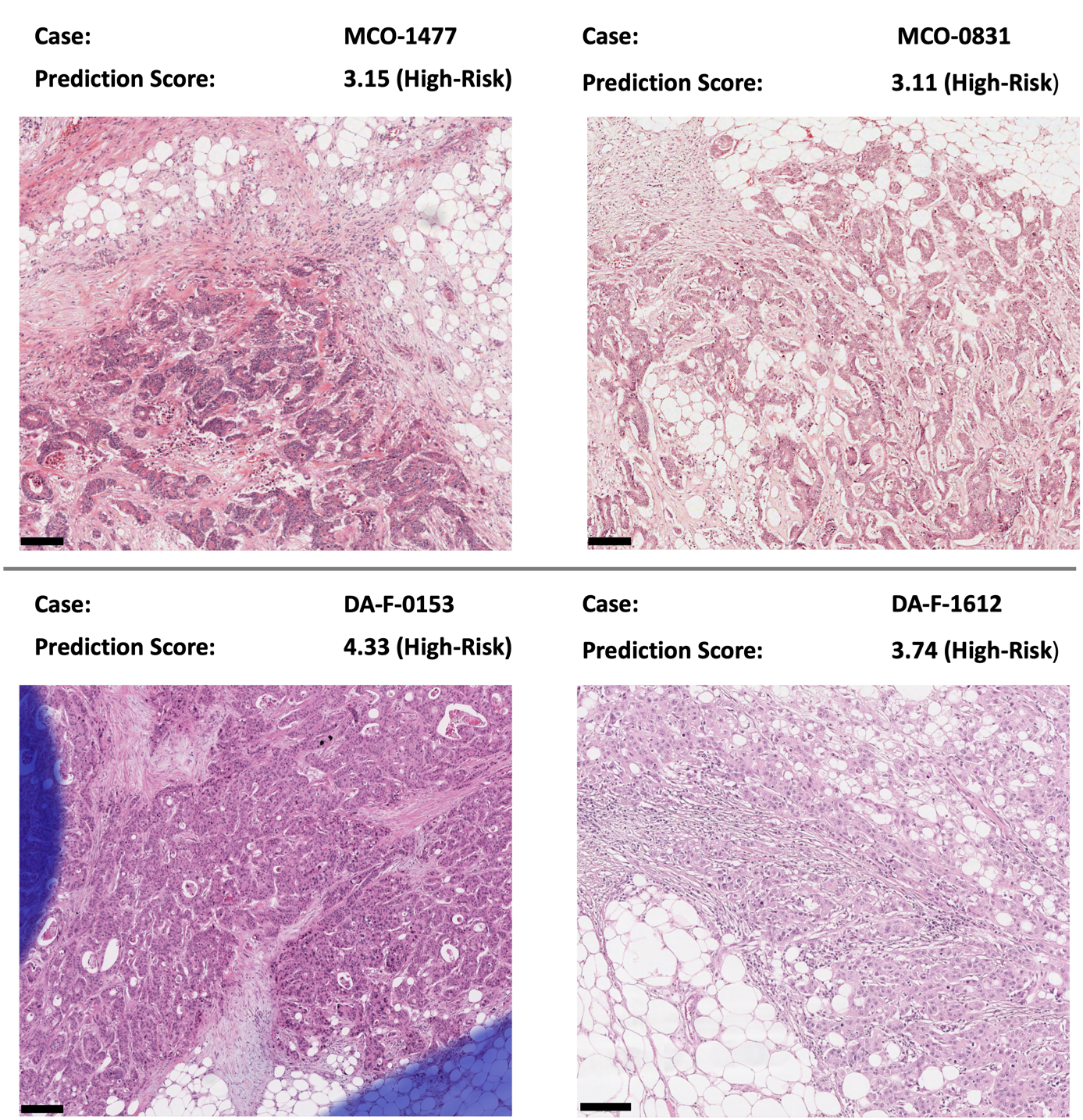


**Figure S9. H&E** **histopathology of CRC cases with the highest DL-based H&E-derived risk scores in the MCO and DACHS cohorts.** The CRCs displayed here show direct tumour–adipocyte interaction at the invasion front (SARIFA positivity) and an infiltrative phenotype. Scale bars, 100 µm. CRC, colorectal cancer; DL, deep learning; SARIFA, Stroma AReactive Invasion Front Areas.

**
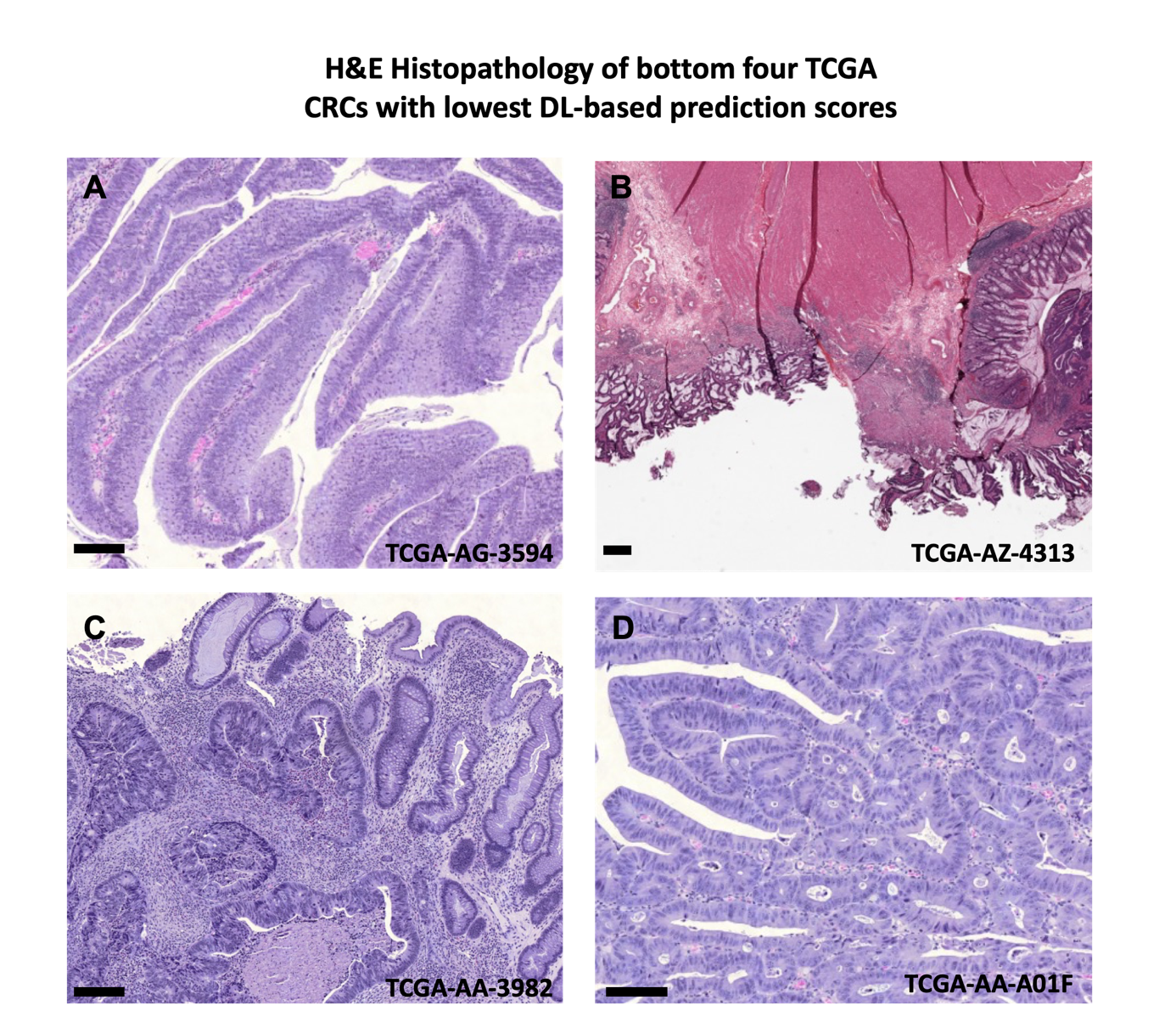
**

**Figure S10. Histopathology of TCGA-CRC cases with the lowest DL-based prediction scores.** (A–D) The cases with the lowest DL-based prediction scores showed often only superficial tumour parts with low-grade adenoma or low-grade early invasive carcinoma with typical gland-forming morphology. Scale bars, 100 µm. The H&E slides are accessible via <https://portal.gdc.cancer.gov> and/or <https://www.cbioportal.org/study/summary?id=coadread_tcga_pub>. CRC, colorectal cancer; DL, deep learning; TCGA, The Cancer Genome Atlas.


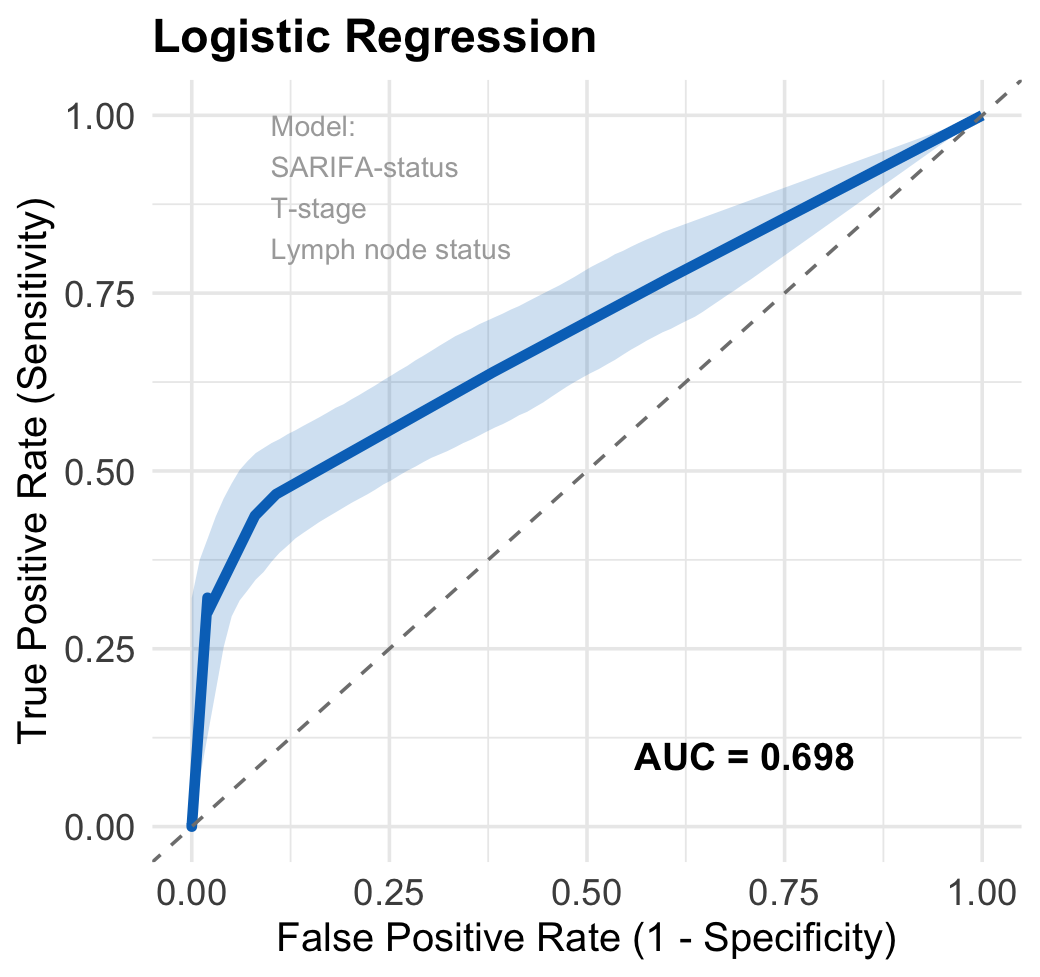


**Figure S11. AUC for logistic regression predicting binary DL-based risk status from SARIFA status, T-stage, and lymph node status as conventional biomarkers.** As T-stage (in TCGA), SARIFA (in DUESSEL and TCGA), and lymph node status (in DUESSEL and TCGA) were associated with DL-based risk groups (low versus high), we built a logistic regression model to determine if those clinicopathological variables could be used as surrogate markers for DL-based risk groups. The distinguishing capability was moderate with an AUC of 0.698. AUC, area under the curve; DL, deep learning; TCGA, The Cancer Genome Atlas.


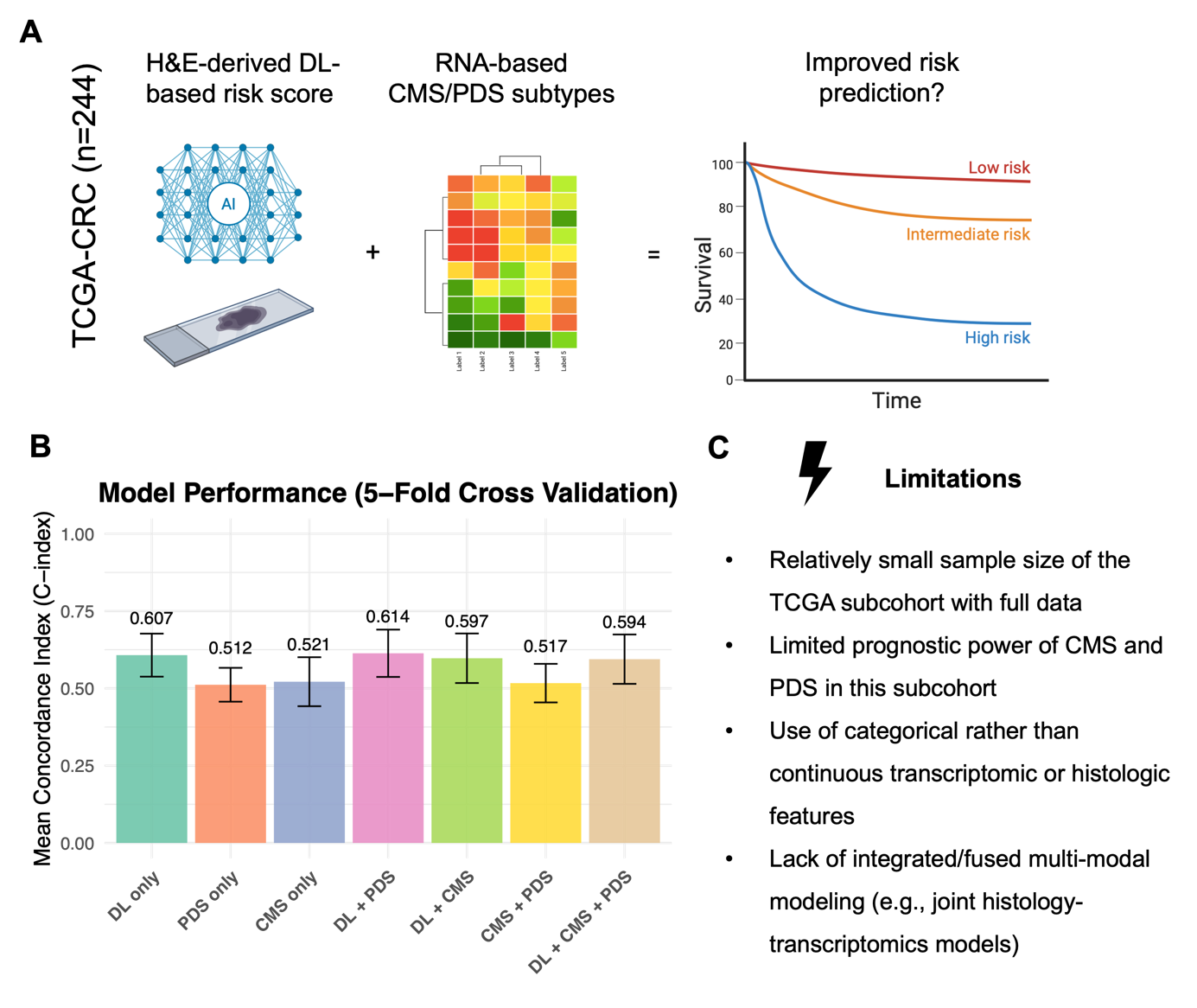
**Figure S12. Association and prognostic value of RNA-based molecular subtypes relative to DL-based risk scores in a TCGA subcohort.** (A) We established CMS and PDS subtypes on RNA-sequencing data of the TCGA-CRC cohort to combine PDS/CMS subgroups with DL-based risk scores into a single prognostic model. (B) Combining DL-based risk assessment and transcriptomic subgroups in a single model did not yield better prognostic performance for this subcohort. (C) Limitations of our approach here include relatively small sample size with full data (DL-based risk scores plus transcriptomic data) and lack of prognostic relevance of CMS and PDS in this TCGA subgroup (CMS: log-rank *p* value = 0.17; PDS: log-rank *p* value = 0.039; DL-based risk scores: log-rank *p* value = 0.0072). There was no difference regarding DL-based risk scores between CMS groups (Kruskal–Wallis *p* value = 0.34) and PDS subtypes (Kruskal–Wallis *p* value = 0.52) in this subcohort. Created in BioRender [Reitsam, N. (2025) <https://BioRender.com/p3ma26v>] and then modified. CMS, consensus molecular subtypes; CRC, colorectal cancer; DL, deep learning; PDS, pathway-derived subtypes; TCGA, The Cancer Genome Atlas.

**
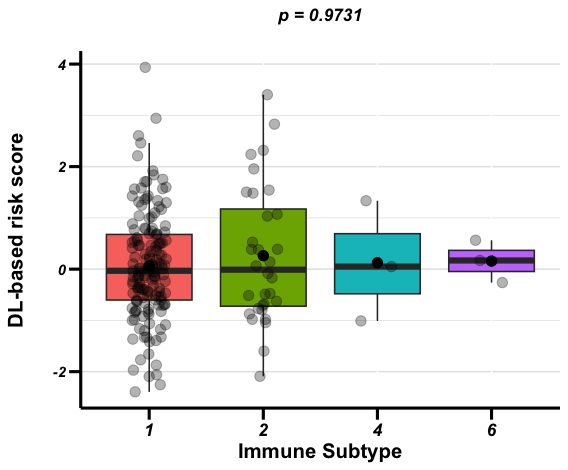
Figure S13. Pan-cancer immune subtypes and DL-based prediction scores in TCGA-CRC.** There were no significant differences between immune subtypes regarding DL-based risk scores. Most CRCs belong to the ‘wound healing’ (1) or ‘IFNγ-dominant’ pan-cancer immune subtype. Subtype 1: wound healing; subtype 2: IFNγ-dominant; subtype 4: lymphocyte-depleted; subtype 6: TGFβ-dominant. CRC, colorectal cancer; DL, deep learning; TCGA, The Cancer Genome Atlas.

**
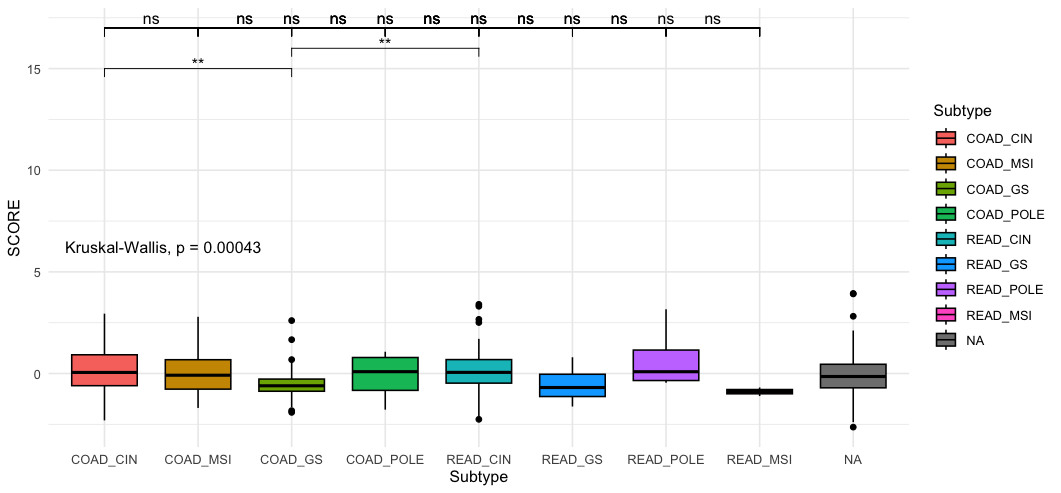
Figure S14. DL-based risk prediction scores in different genetically defined CRC subgroups of TCGA.** Pairwise comparisons of DL-based risk scores across genetically defined subtypes of COAD and READ revealed that CIN-COADs and CIN-READs had significantly higher DL-based prediction scores compared with GS-COADs. Subgroups of COAD, colonic adenocarcinoma, and READ, rectal adenocarcinoma. CIN, chromosomal-unstable; MSI, microsatellite-unstable; GS, genomically stable; POLE, polymerase epsilon-mutated.

**Figure 15.** **DL-based H&E-inferred risk scores and their association with the aggressive subgroup of *BRAF*-mutant/MSS CRCs.** (A) DACHS cohort; (B) MCO cohort. *BRAF*-mutant/MSS CRCs show significantly higher DL-based risk scores than do non-*BRAF*-mutant/MSS CRCs (both *p* < 0.01). *p* values of Wilcoxon tests are displayed. CRC, colorectal cancer; DL, deep learning; MSS, microsatellite-stable; TCGA, The Cancer Genome Atlas.

*
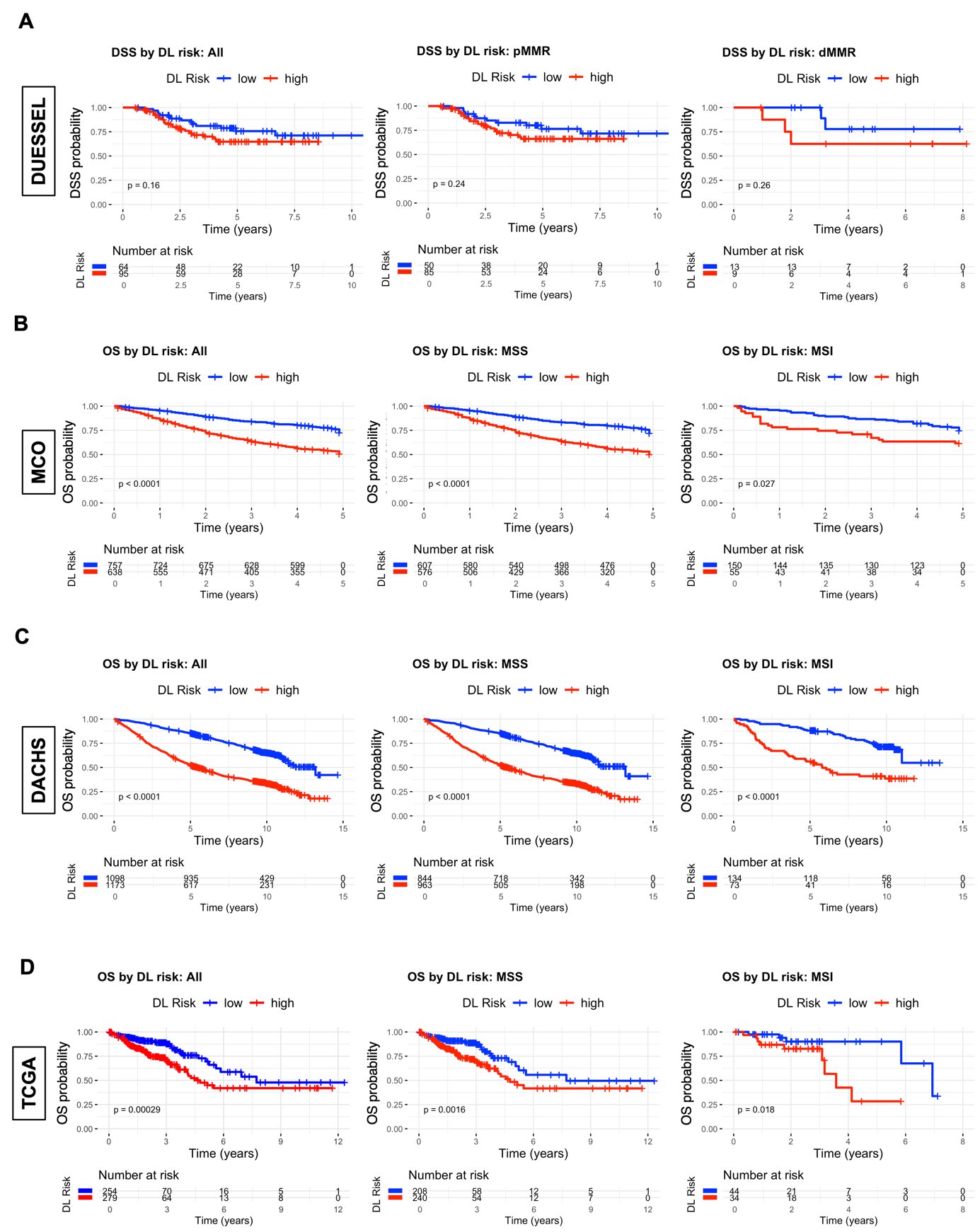
*

**Figure S16.** **Survival curves of CRC patients stratified by MSI/MSS status.** (A) Duesseldorf cohort; (B) MCO cohort; (C) DACHS cohort; (D) TCGA cohort. *p* values of log-rank tests are displayed. CRC, colorectal cancer; DL, deep learning; DSS, disease-specific survival; MMR, mismatch repair status (proficient or deficient); MSI, microsatellite-unstable; MSS, microsatellite-stable; OS, overall survival; TCGA, The Cancer Genome Atlas.

*
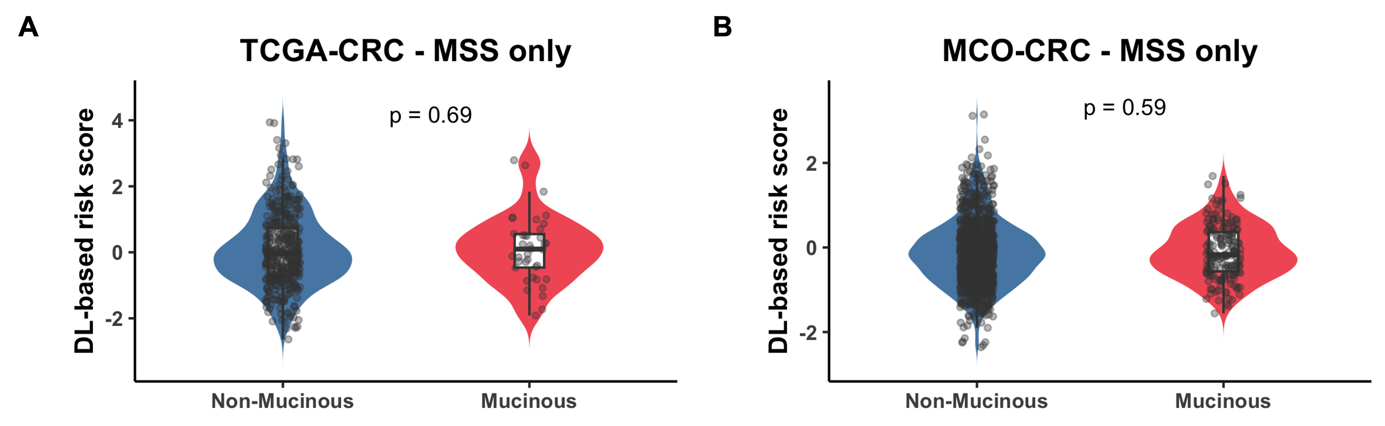
*

**Figure S17.** **DL-based H&E-inferred risk scores and their association with mucinous histology in the MSS subgroup.** (A) TCGA-CRC; (B) MCO-CRC. Mucinous CRCs do not show significantly higher DL-based risk scores than do non-mucinous CRCs in the MSS subgroup (both *p* > 0.05). *p* values of Wilcoxon tests are displayed. CRC, colorectal cancer; DL, deep learning; MSS, microsatellite-stable; TCGA, The Cancer Genome Atlas.

**Supplementary tables**

**Table S1.** Relationship between DL-based risk group and clinicopathological features (DUESSEL).

| **Duesseldorf cohort** |  | **All cases** |  | **DL-based low-risk** |  | **DL-based high-risk** |  |  |
| --- | --- | --- | --- | --- | --- | --- | --- | --- |
|  |  |  | in % |  | in % |  | in % |  |
|  |  | *n* = 159 | 40 | *n =*64 |  | *n* = 95 | 60 | ***p* value** |
| **Median age (range), yrs** | | 69 (45–88) |  | 70 (53–87) |  | 68 (45–88) |  | 0.2 |
| **Sex** |  |  |  |  |  |  |  | 0.6 |
|  | Male | 67 | 42 | 25 | 39 | 42 | 44 |  |
|  | Female | 92 | 58 | 39 | 61 | 53 | 56 |  |
| **Tumour location** | |  |  |  |  |  |  | 0.4 |
|  | Colon | 102 | 64 | 38 | 59 | 64 | 67 |  |
|  | Rectum | 57 | 36 | 26 | 41 | 31 | 33 |  |
| **pT category** |  |  |  |  |  |  |  | 0.9 |
|  | pT1/pT2 | 47 | 30 | 18 | 28 | 29 | 31 |  |
|  | pT3/pT4 | 112 | 70 | 46 | 72 | 66 | 69 |  |
| **pN category** |  |  |  |  |  |  |  | **0.008** |
|  | pN0 | 101 | 64 | 49 | 77 | 52 | 55 |  |
|  | pN1/pN2 | 58 | 36 | 15 | 23 | 43 | 45 |  |
| **Lymphovascular invasion** |  |  |  |  |  |  |  | 0.064 |
|  | Absent | 110 | 69 | 45 | 70 | 65 | 68 |  |
|  | Present | 49 | 31 | 19 | 30 | 30 | 32 |  |
| **Grading** |  |  |  |  |  |  |  | 0.2 |
|  | Low | 121 | 76 | 53 | 83 | 68 | 72 |  |
|  | High | 38 | 24 | 11 | 17 | 27 | 28 |  |
| **MMR status** |  |  |  |  |  |  |  | 0.062 |
| *n =*2 missing | dMMR | 22 | 14 | 13 | 21 | 9 | 10 |  |
|  | pMMR | 135 | 86 | 50 | 79 | 85 | 90 |  |
| **SARIFA status** |  |  |  |  |  |  |  | **<0.001** |
|  | Negative | 124 | 24 | 60 | 94 | 64 | 67 |  |
|  | Positive | 35 | 76 | 4 | 6 | 31 | 33 |  |
| **Proportion of tumour** |  |  |  |  |  |  |  | 0.9 |
|  | Low | 26 | 24 | 11 | 26 | 15 | 23 |  |
|  | High | 81 | 76 | 31 | 74 | 50 | 77 |  |
| *p* values that are statistically significant are highlighted in bold. | | | | |  |  |  |  |
| MMR- and SARFIFA-status with Fisher’s test as sample size were small in subgroups; for age: Wilcoxon test; for all other categorical variables: *χ*^2^ test | | | | | | | | |
| SARIFA, Stroma AReactive Invasion Front Areas; pT, depth of invasion; pN, lymph node status; PoT, proportion of tumour; MMR, mismatch repair status (proficient or deficient). | | | | | | | | |

**Table S2.** Relationship between DL-based risk group and clinicopathological features (TCGA-COAD/READ).

| **TCGA cohort** |  | **All cases** |  | **DL-based low-risk** |  | **DL-based high-risk** |  |  |
| --- | --- | --- | --- | --- | --- | --- | --- | --- |
|  |  |  | in % |  | in % |  | in % |  |
|  |  | *n* = 533 |  | *n* = 254 |  | *n* = 279 |  | *p* value |
| **Median age (range), yrs** | | 67 (31–90) |  | 67 (31–90) |  | 67 (34–90) |  | 0.15 |
| **Sex** |  |  |  |  |  |  |  | 0.6 |
|  | Male | 278 | 52 | 129 | 51 | 149 |  |  |
|  | Female | 255 | 48 | 125 | 49 | 130 |  |  |
| **Tumour location** | |  |  |  |  |  |  | 0.8 |
|  | Colon | 397 | 74 | 191 | 75 | 206 | 74 |  |
|  | Rectum | 136 | 26 | 63 | 25 | 73 | 26 |  |
| **T stage** |  |  |  |  |  |  |  | **<0.001** |
|  | TIS/T1/T2 | 115 | 22 | 77 | 30 | 38 |  |  |
|  | T3/T4 | 418 | 78 | 177 | 70 | 241 |  |  |
| **Nodal status** |  |  |  |  |  |  |  | **<0.001** |
| *n =*1 *with NX (no nodal status available)* | negative | 302 | 57 | 171 | 67 | 131 | 47 |  |
|  | positive | 230 | 43 | 83 | 33 | 147 | 53 |  |
| **MSI status** |  |  |  |  |  |  |  | 0.13 |
| *MSIsensorScore <3.5* | MSS | 448 | 85 | 208 | 83 | 240 | 88 |  |
| *MSIsensorScore ≥M3.5* | MSI | 78 | 15 | 44 | 17 | 34 | 12 |  |
| **SARIFA status** |  |  |  |  |  |  |  | **<0.001** |
| *n =*192 | Negative | 128 | 67 | 79 | 91 | 49 | 47 |  |
|  | Positive | 64 | 33 | 8 | 9 | 56 | 53 |  |
| **TAF** |  |  |  |  |  |  |  | **<0.001** |
| *n =*192 | Absent | 88 | 46 | 55 | 63 | 33 | 31 |  |
|  | Present | 104 | 54 | 32 | 37 | 72 | 69 |  |
| **Mucinous differentiation** | |  |  |  |  |  |  | 0.97 |
| *According to TCGA metadata* | Yes | 70 | 13 | 34 | 13 | 36 | 13 |  |
|  | No | 463 | 87 | 220 | 87 | 243 | 87 |  |
| **Race category** |  |  |  |  |  |  |  | **0.015** |
|  | American Indian or Alaska Native | 1 | 0.2 | 0 | 0 | 1 | 0.4 |  |
|  | Asian | 12 | 2 | 1 | 0.5 | 11 | 4.0 |  |
|  | Black or African American | 62 | 12 | 24 | 9.5 | 38 | 13.6 |  |
|  | White | 268 | 50 | 127 | 50 | 141 | 51 |  |
|  | NA | 190 | 36 | 102 | 40 | 88 | 32 |  |
| *p* values that are statistically significant are highlighted in bold. | | | | |  |  |  |  |
| For age: Wilcoxon test; for categorical variables: *χ*^2^ test (except for race category: Fisher test). | | | | | | | | |
| COAD, colonic adenocarcinoma; READ, rectal adenocarcinoma; SARIFA, Stroma AReactive Invasion Front Areas; MSI, microsatellite instability; TCGA, The Cancer Genome Atlas; NX, not available. | | | | | | | | |

**Table S3.** Relationship between DL-based risk group and clinicopathological features (DACHS).

| **DACHS cohort** |  | **All cases** |  | **DL-based low-risk** |  | **DL-based high-risk** |  |  |
| --- | --- | --- | --- | --- | --- | --- | --- | --- |
|  |  |  | in % |  | in % |  | in % |  |
|  |  | *n* = 2,271 |  | *n* = 1,098 |  | *n* = 1,173 |  | *p* value |
| **Median age (range), yrs** | | 69 (30–96) |  | 69 (33–94) |  | 70 (30–96) |  | **<0.001** |
| **Sex** |  |  |  |  |  |  |  | 0.7 |
|  | Male | 1,332 | 59 | 459 | 58 | 693 | 59 |  |
|  | Female | 939 | 41 | 639 | 42 | 480 | 41 |  |
| **T-stage** |  |  |  |  |  |  |  | **<0.001** |
| *n =*50 *with TX* | T1/T2 | 514 | 23 | 383 | 38 | 108 | 9 |  |
|  | T3/T4 | 1,707 | 77 | 670 | 62 | 1037 | 91 |  |
| **Nodal status** |  |  |  |  |  |  |  | **<0.001** |
| *n =*54 *with NA* | Negative | 1,202 | 54 | 711 | 66 | 491 | 43 |  |
|  | Positive | 1,015 | 46 | 362 | 34 | 653 | 57 |  |
| **MSI status** |  |  |  |  |  |  |  | **<0.001** |
| *n =*257 *with NA* | Non-MSI-H | 207 | 10 | 134 | 14 | 73 | 7 |  |
|  | MSI-H | 1,807 | 90 | 844 | 86 | 963 | 93 |  |
| ***BRAF*** |  |  |  |  |  |  |  | 0.55 |
| *n =*219 *with NA* | WT | 1,904 | 93 | 67 | 7 | 81 | 8 |  |
|  | MT | 148 | 7 | 930 | 93 | 974 | 92 |  |
| ***KRAS*** |  |  |  |  |  |  |  | 0.89 |
| *n =*225 *with NA* | WT | 1,378 | 67 | 666 | 68 | 712 | 67 |  |
|  | MUT | 668 | 33 | 320 | 32 | 348 | 33 |  |
| **Grade group** |  |  |  |  |  |  |  | **<0.001** |
|  | Low | 1,504 | 74 | 802 | 81 | 702 | 66 |  |
|  | High | 543 | 26 | 190 | 19 | 353 | 34 |  |
| *p* values that are statistically significant are highlighted in bold. | | | | |  |  |  |  |
| For age: Wilcoxon test; for categorical variables: *χ*^2^ test. | | | | | | | | |
| MSI-H, microsatellite instability-high; WT, wild type; MUT, mutant. | | | | | | | | |
| TX indicates no T stage available; NA indicates not available. | | | | | | | | |

**Table S4.** Relationship between DL-based risk group and clinicopathological features (MCO).

| **MCO cohort** |  | **All cases** |  | **DL-based low-risk** |  | **DL-based high-risk** |  |  |
| --- | --- | --- | --- | --- | --- | --- | --- | --- |
|  |  |  | in % |  | in % |  | in % |  |
|  |  | *n* = 1,395 |  | *n =*757 |  | *n* = 638 |  | *p* value |
| **Median age (range), yrs** | | 69 (24–99) |  | 69 (27–99) |  | 69 (24–99) |  | 0.89 |
| **Sex** |  |  |  |  |  |  |  | 0.07 |
|  | Male | 767 | 55 | 399 | 53 | 368 | 57 |  |
|  | Female | 628 | 45 | 358 | 47 | 270 | 43 |  |
| **T-stage** |  |  |  |  |  |  |  | **<0.001** |
| *´* | T1/T2 | 342 | 25 | 277 | 37 | 65 | 90 |  |
|  | T3/T4 | 1,053 | 75 | 480 | 63 | 573 | 10 |  |
| **Nodal status** |  |  |  |  |  |  |  | **<0.001** |
|  | Negative | 796 | 57 | 506 | 67 | 290 | 45 |  |
|  | Positive | 599 | 42 | 251 | 22 | 348 | 55 |  |
| **MSI status** |  |  |  |  |  |  |  | **<0.001** |
| *n =*7 *not available* | Stable | 1,183 | 85 | 607 | 80 | 576 | 90 |  |
|  | Unstable | 205 | 15 | 150 | 20 | 55 | 10 |  |
| ***BRAF*** |  |  |  |  |  |  |  | 0.11 |
| *n =*7 *not available* | WT | 1,228 | 88 | 658 | 87 | 63 | 89 |  |
|  | MUT | 160 | 12 | 97 | 13 | 570 | 11 |  |
| ***KRAS*** |  |  |  |  |  |  |  | 0.80 |
| *n =*5 *not available* | WT | 961 | 69 | 520 | 69 | 570 | 70 |  |
|  | MUT | 429 | 31 | 236 | 31 | 193 | 30 |  |
| **Grade group** |  |  |  |  |  |  |  | **0.004** |
| *n =*25 *not available* | Low | 1,180 | 86 | 657 | 89 | 523 | 83 |  |
|  | High | 190 | 14 | 84 | 11 | 106 | 17 |  |
| *p* values that are statistically significant are highlighted in bold. | | | | |  |  |  |  |
| For age: Wilcoxon test; for categorical variables: *χ*^2^ test. | | | | | | | | |
| WT, wild type; MUT, mutant. | | | | | | | | |

**Table S5.** Relationship between DL-based risk group and metastasis.

|  |  | **Proportions** | |  |
| --- | --- | --- | --- | --- |
| **Cohort** | **DL-based risk** | **No metastasis** | **Metastasis** | ***p* value of *χ*^2^ or Fisher’s exact test** |
| **DACHS** |  |  |  |  |
| *n* = 1,682 | Low | 0.94 | 0.60 | **<0.001** |
|  | High | 0.69 | 0.31 |  |
| **MCO** |  |  |  |  |
| *n* = 1,395 | Low | 0.95 | 0.05 | **<0.001** |
|  | High | 0.79 | 0.21 |  |
| **DUESSEL** |  |  |  |  |
| *n* = 159 | Low | 1.00 | 0.0 | 1 |
|  | High | 0.99 | 0.01 |  |
| **TCGA** |  |  |  |  |
| *n* = 459 | Low | 0.95 | 0.05 | **<0.001** |
|  | High | 0.81 | 0.19 |  |
| *p* values that are statistically significant are highlighted in bold. | | | | |
| Only patients with information on metastasis were included. | | | | |
| In the DUESSEL cohort, there were only two cases with metastases. | | | | |

**Table S6.** Potential antibody–drug conjugate (ADC) and radiopharmaceutical therapy (RPT) targets in colorectal cancer.

| **Gene** | **Therapeutic group** | **Explanation** | **Reference** |
| --- | --- | --- | --- |
| *ERBB3* | ADC | HER3 | doi.org/10.1158/1535-7163.MCT-19-0452 |
| *GOLM1* | ADC |  | doi.org/10.1007/s11523-019-00667-z |
| *EGFR* | ADC |  | doi.org/10.18632/oncotarget.26002 |
| *TACSTD2* | ADC |  | doi.org/10.1038/s41416-023-02180-7; doi.org/10.1038/s41698-024-00584-z |
| *NECTIN4* | ADC |  | doi.org/10.1038/s41416-023-02180-7 |
| *ERBB2* | ADC |  | doi:10.1001/jamaoncol.2021.8196 |
| *DLL3* | ADC |  | doi.org/10.1158/2767-9764.CRC-24-0501 |
| *PTK7* | ADC |  | doi: 10.1158/1535-7163.MCT-23-0164 |
| *F3* | ADC | CD142, Tissue Factor | doi:10.3390/pharmaceutics15082160; doi: 10.3390/ijms241411585 |
| *FOLR1* | ADC |  | doi: 10.1016/j.humpath.2007.09.013 |
| *SLC39A6* | ADC | LIV1 | doi.org/10.1038/s41598-025-03713-1 |
| *CEACAM1* | ADC |  | doi: 10.1007/s10555-013-9444-6 |
| *MSLN* | ADC |  | doi.org/10.1038/s41417-024-00816-1 |
| *MET* | ADC |  | doi:10.3748/wjg.v21.i12.3706 |
| *HHLA2* | ADC |  | doi.org/10.1158/1538-7445.AM2025-7336 |
| *CD276* | ADC |  | doi.org/10.1038/s41591-025-03600-2 |
| *FAP* | RPT |  | doi: 10.1245/s10434-024-16593-y; doi.org/10.3389/pore.2023.1611163 |
| *ITGAV* | RPT |  | doi:10.7150/thno.39203 |
| *GPA33* | RPT |  | doi.org/10.2967/jnumed.124.267685 |
| *ITGA4* | RPT |  | doi: 10.1158/1535-7163.MCT-24-0370 |
| *SLC3A2* | ADC |  | doi.org/10.1186/s13046-023-02784-0 |
| *EREG* | ADC |  | doi.org/10.1158/0008-5472.CAN-24-0798 |
| *ADGRG1* | ADC |  | doi:10.1038/s41416-023-02192-3 |
| *CLDN18* | ADC |  | doi: 10.1016/j.modpat.2025.100712 |
| *CCR5* | RPT |  | doi:10.1080/2162402X.2019.1626193; https://cds.cern.ch/record/2902594?ln = en |
| *CDH17* | ADC |  | https://trial.medpath.com/news/70fd3a5156e6c0ec/keymed-biosciences-advances-novel-cdh17-targeted-adc-for-gastrointestinal-cancers-with-ind-approval-in-china |
| *ADAM9* | ADC |  | doi.org/10.1158/1535-7163.MCT-21-0915 |
| *GRPR* | RPT |  | doi.org/10.1039/D0BM01432J |
| *CDCP1* | ADC |  | https://innovation.weill.cornell.edu/industry-investors-partners/technology-portfolio/cdcp1-novel-therapeutic-target-cancer-therapy |
| *SERPINE1* | Chemo-resistant | RESIST-M1 | doi.org/10.1038/s41419-025-07855-y |
| *SMARCD3* | Chemo-resistant | RESIST-M1 | doi.org/10.1038/s41419-025-07855-y |
| *SC5D* | Chemo-responsive | RESIST-M2 | doi.org/10.1038/s41419-025-07855-y |
| *FDPS* | Chemo-responsive | RESIST-M2 | doi.org/10.1038/s41419-025-07855-y |
| *MVD* | Chemo-responsive | RESIST-M2 | doi.org/10.1038/s41419-025-07855-y |
| *HMGCS1* | Chemo-responsive | RESIST-M2 | doi.org/10.1038/s41419-025-07855-y |
| *HMGCR* | Chemo-responsive | RESIST-M2 | doi.org/10.1038/s41419-025-07855-y |
| *CYP5A1A1* | Chemo-responsive | RESIST-M2 | doi.org/10.1038/s41419-025-07855-y |
| *ACAT2* | Chemo-responsive | RESIST-M2 | doi.org/10.1038/s41419-025-07855-y |
| *ANKRD1* | Chemo-resistant |  | doi.org/10.1038/bjc.2011.505 |
| *ANKRD40* | Chemo-resistant |  | doi.org/10.1038/bjc.2011.505 |
| *CIRBP* | Chemo-resistant |  | doi.org/10.1038/bjc.2011.505 |
| *CREB1* | Chemo-resistant |  | doi.org/10.1038/bjc.2011.505 |
| *THY1* | Chemo-responsive |  | DOI: 10.1016/j.xcrm.2024.101661 |
| *BUB1* | Chemo-responsive |  | DOI: 10.1016/j.xcrm.2024.101661 |
| *DEPDC1* | Chemo-responsive |  | DOI: 10.1016/j.xcrm.2024.101661 |
| *STON1* | Chemo-responsive |  | DOI: 10.1016/j.xcrm.2024.101661 |
| *ATAD2* | Chemo-responsive |  | DOI: 10.1016/j.xcrm.2024.101661 |
| *HSD17B2* | Chemo-responsive |  | DOI: 10.1016/j.xcrm.2024.101661 |
| *TPX2* | Chemo-responsive |  | DOI: 10.1016/j.xcrm.2024.101661 |
| *AURKB* | Chemo-responsive |  | DOI: 10.1016/j.xcrm.2024.101661 |
| *CYR61* | Chemo-responsive |  | DOI: 10.1016/j.xcrm.2024.101661 |
| *FAM84A* | Chemo-responsive |  | DOI: 10.1016/j.xcrm.2024.101661 |
| *ITGB6* | RPT |  | doi: 10.1136/jitc-2021-003465 |
| *ADGRE5* | ADC |  | https://doi.org/10.1016/j.celrep.2023.113374 |
